# Chloroplast Auxin Efflux Mediated by ABCB28 and ABCB29 Fine-Tunes Salt and Drought Stress Responses in *Arabidopsis*

**DOI:** 10.3390/plants13010007

**Published:** 2023-12-19

**Authors:** Prashanth Tamizhselvan, Sharmila Madhavan, Christian Constan-Aguilar, Eman Ryad Elrefaay, Jie Liu, Aleš Pěnčík, Ondřej Novák, Albert Cairó, Mónika Hrtyan, Markus Geisler, Vanesa Beatriz Tognetti

**Affiliations:** 1 Mendel Centre for Plant Genomics and Proteomics, Central European Institute of Technology (CEITEC), Masaryk University, Kamenice 5, CZ-625 00 Brno, Czech Republic; prashanthts1987@gmail.com (P.T.); sharmilamadhavan712@gmail.com (S.M.); constzzone@gmail.com (C.C.-A.); 430783@mail.muni.cz (E.R.E.); albert.calzada@ceitec.muni.cz (A.C.); monika.hrtyan@ist.ac.at (M.H.); 2 Department of Biology, University of Fribourg, CH-1700 Fribourg, Switzerland; happyliujie1234@163.com (J.L.); markus.geisler@unifr.ch (M.G.); 3 Laboratory of Growth Regulators, Institute of Experimental Botany, The Czech Academy of Sciences, & Faculty of Science, Palacký University, Šlechtitelů 27, CZ-783 71 Olomouc, Czech Republic; alespencik@seznam.cz (A.P.); ondrej.novak@upol.cz (O.N.)

**Keywords:** auxin, hormone transport, ABC transporter, stress, photosynthesis, drought, salinity

## Abstract

Photosynthesis is among the first processes negatively affected by environmental cues and its performance directly determines plant cell fitness and ultimately crop yield. Primarily sites of photosynthesis, chloroplasts are unique sites also for the biosynthesis of precursors of the growth regulator auxin and for sensing environmental stress, but their role in intracellular auxin homeostasis, vital for plant growth and survival in changing environments, remains poorly understood. Here, we identified two ATP-binding cassette (ABC) subfamily B transporters, ABCB28 and ABCB29, which export auxin across the chloroplast envelope to the cytosol in a concerted action in vivo. Moreover, we provide evidence for an auxin biosynthesis pathway in *Arabidopsis thaliana* chloroplasts. The overexpression of ABCB28 and ABCB29 influenced stomatal regulation and resulted in significantly improved water use efficiency and survival rates during salt and drought stresses. Our results suggest that chloroplast auxin production and transport contribute to stomata regulation for conserving water upon salt stress. ABCB28 and ABCB29 integrate photosynthesis and auxin signals and as such hold great potential to improve the adaptation potential of crops to environmental cues.

## 1. Introduction

Photosynthesis is among the first processes negatively affected by environmental cues and it directly determines plant cell fitness and, ultimately, crop yield [1]. Primarily sites of photosynthesis, chloroplasts are unique sites also for the biosynthesis of precursors and end products involved in many crucial metabolic pathways. Key steps in biosynthesizing hormones occur in the chloroplasts and might act as chloroplast signals [1,2]. These and other more specific substances must be exported to the rest of the cell. Therefore, chloroplasts’ proper functioning under stress vitally depends on the orchestrated regulation of a variety of transport systems located at the chloroplast membranes. Within the hormonal network, auxin functions as master regulator in plant developmental plasticity, a pivotal response for acclimating to a changing environment [1]. We have previously described that auxin conjugation in *Arabidopsis thaliana* plays a role in osmotic stress adaptation, and in the protection of photosynthesis in young tissues [3]. Although the earlier findings were also consistent with a link between the auxin pathway and photosynthesis [1], the molecular mechanisms are not yet known. Despite the fact that different plant species may have unique strategies, indole-3-acetic acid (IAA), the most common auxin class, is thought to be most likely synthesized in cytosol [4,5,6,7,8,9,10,11]. However, notwithstanding early evidence showing the accumulation of IAA in the chloroplasts [12,13] and the ability of chloroplasts to synthesize IAA [8,12,14,15,16], almost nothing is known about IAA metabolism and transport in chloroplasts or their roles for plant growth and development.

ATP-binding cassette (ABC) proteins form a large and ubiquitous superfamily of transporters with a wide range of substrate specificities and participate in such essential physiological processes as development, growth, nutrient acquisition, and stress responses [17,18,19,20]. The more than 130 members of the *Arabidopsis* ABC protein family are classified into eight subfamilies: ABCA–ABCG and ABCI [21]. Most membrane ABC proteins consist of two similar transmembrane domains (TMDs) and two similar nucleotide binding domains (NBDs). These are termed “full-size” ABCs. So-called “half-size” ABCs, meanwhile, have one TMD and one NBD, and to become functional, they homo- or heterodimerize. The NBDs are involved in Mg–ATP binding and hydrolysis to drive active transport [20]. Although several proteins of each subfamily have been characterized, the vast majority remain uncharacterized [19]. The *Arabidopsis* ABCB subfamily consists of 21 full-size and 7 half-size proteins [21]. The full-size ABCBs described to date are localized at the plasma membrane, and several of them (i.e., ABCB1, 4, 6, 15–18, 19–22) are involved in the transport of auxin [22,23]. Half-size ABCBs are present in the chloroplasts [24,25,26], mitochondria [20], and on the vacuole [20]. The chloroplast inner envelope membrane (IE) harbors three ABCBs—ABCB25, ABCB26, and ABCB28 [24,25,27,28]. ABCB25 also localizes in the mitochondrial inner membrane with the NBDs facing the matrix [29,30,31], and has been shown to export transport glutathione–cadmium or cadmium–sulfur complexes [32]. ABCG7 was found in the outer envelope membrane (OE) of the chloroplast [25,33]. Finally, ABCB29 was identified in chloroplast whole envelope membranes [28,34], but its intraorganellar location within the chloroplast is not known.

In spite of substantial progress in our understanding of IAA action, there are still gaps in our knowledge about the organelle-specific biosynthesis and the distribution of IAA. To shed light on auxin homeostasis at the subcellular level, we investigated the link between chloroplast and auxin homeostasis and the functions of ABCB28 and ABCB29. In this study, we demonstrate that ABCB28 and ABCB29 mediate the transport of IAA across the chloroplast envelope and provide evidence for an IAA biosynthesis pathway in *Arabidopsis* chloroplasts. Moreover, based on the analysis of transgenic plants, we determined the role of both ABCB transporters in the osmotic stress response. The results constitute a breakthrough in the field of intracellular auxin transport and its vital role for plant survival in changing and hostile environments. 

## 2. Results

### 2.1. Cell-Specific Gene Expression and Subcellular Localization of ABCB28 and ABCB29 

*Arabidopsis* ABCB28 and ABCB29 were identified in proteomics studies of the chloro-plast envelope membranes and the AT_CHLORO database [24,26,27,34]. Moreover, TargetP-2.0 (https://services.healthtech.dtu.dk/services/TargetP-2.0/ (accessed on 25 October 2023) [35] predicted the plastid transit peptide signals at the N-termini of the ABCB28 and ABCB29 protein sequences of 65 and 58 amino acids, respectively (Figure S1a). The chloroplast transit peptides guide the import of nuclear-encoded precursor proteins from the cytoplasm into the chloroplasts, and are subsequently removed [36]. Genevestigator analysis [37] showed that the ABCB28 and ABCB29 genes are preferentially expressed in the rosette, and also in the root apical meristem in the case of *ABCB28*. The higher expression of *ABCB29* compared to that of *ABCB28* was observed in leaf primordia and in juvenile and adult leaves, and higher expression of ABCB28 was induced in senescent leaves (Figure S1b).

**Figure 1:**
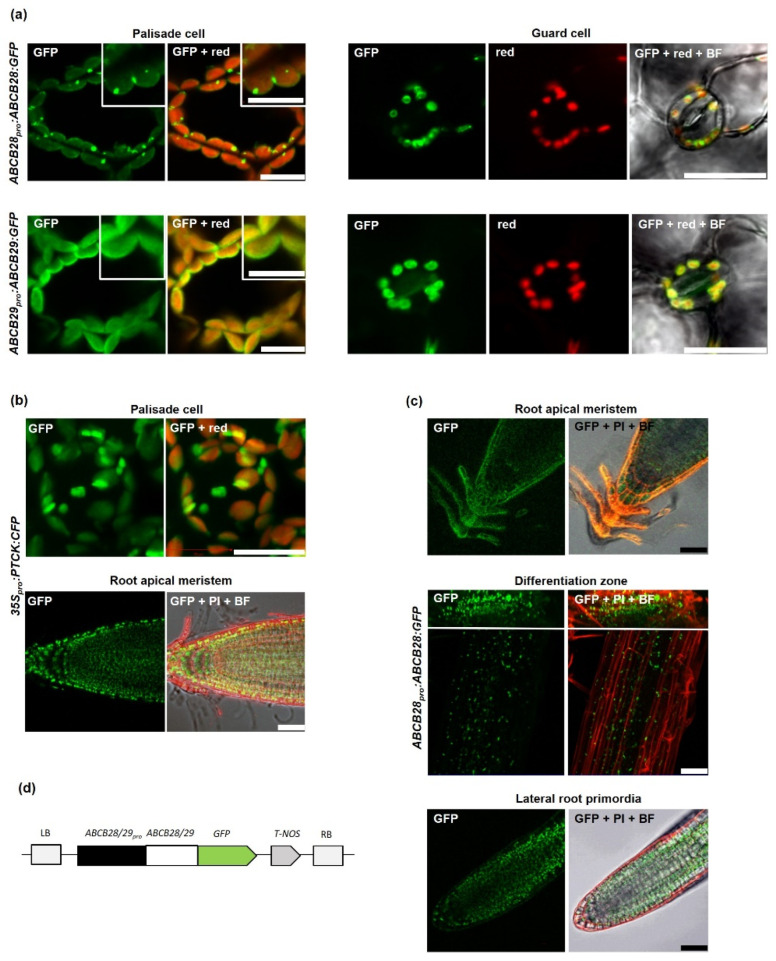
Intracellular localization and tissue expression pattern of ABCB28 and ABCB29 using confocal laser scanning microscopy. (**a**) Subcellular localization of ABCB28-GFP and ABCB29-GFP in chloroplasts of palisade mesophyll cells and guard cells from leaves excised from *ABCB28_pro_:ABCB28:GFP* and *ABCB29_pro_:ABCB29:GFP* transgenic plants. Scale bars: 20 µm. Insets show close-ups of chloroplasts in mesophyll cells. Scale bars: 5 µm. Chlorophyll autofluorescence is shown as red; (**b**) confocal images of leaf palisade mesophyll and propidium iodide (PI)-stained (red) root apical meristem cells from the stromal marker *35S_pro_:PTCK:CFP* plants. Scale bars: 20 µm. Chlorophyll autofluorescence is shown as red; (**c**) plastid localization of ABCB28-GFP in cells from root apical meristem, differentiation root region, and lateral root primordia from *ABCB28_pro_:ABCB28:GFP* PI-stained roots; (**d**) schematic representation of the constructs used for the generation of transgenic lines. *T-NOS*; nopaline synthase terminator and poly(A) signal. Scale bars: 20 µm. (**a**–**c**) BF, bright field. Presented are representative images from two experiments and from seedlings 21 days old.

Therefore, to confirm the localization of ABCB28 and ABCB29 in vivo, we generated *Arabidopsis* plants expressing ABCB28 or ABCB29 proteins fused at their C-terminus to the green fluorescent protein (GFP) or cyan fluorescence protein (CFP), either under the control of their own promoters (*ABCB28_pro_:ABCB28:GFP*, *ABCB29_pro_:ABCB29:GFP*) or under the constitutive cauliflower mosaic virus *35S* promoter (*35S_pro_:ABCB28:GFP*, *35S_pro_:ABCB29:CFP*) in the wild-type (WT) background. Confocal laser scanning microscopy (CLSM) verified ABCB28-GFP and ABCB29-CFP/GFP signals being distributed around the plastid periphery of the leaf mesophyll cells and of the stomata cells in all the transgenic lines. Punctate protrusions and a patchy distribution of fluorescence signals were also detected on the surface of chloroplasts from the *ABCB28_pro_:ABCB28:GFP*, *35S_pro_:ABCB28:GFP*, and *35S_pro_:ABCB29:CFP* plants (Figure 1a and Figure S2a). However, in *Arabidopsis* leaves transiently expressing ABCB28-GFP or ABCB29-CFP, a uniform distribution of the fluorescence signal was observed around the plastid envelope of the mesophyll cells (Figure S3). High expression levels in transient expression assays of OE and IE proteins can lead to alterations in the shape of the respective membrane and might be considered artifacts of fusion protein overexpression [38,39]. Similarly, localized protein patches were observed in several stable transgenic plants overexpressing OE and IE fluorescence protein fusions [40,41,42,43] without altering growth and development [41,44]. Moreover, the effect was proposed to be a product of the GFP construct [44]. Therefore, the formation of punctate protrusions of ABCB28-GFP signals in the *ABCB28_pro_:ABCB28:GFP* line might suggests that ABCB28 is highly expressed in the leaves. Alternatively, ABCB28 might actually localize as small domains to increase its outreach and interactivity with other cytosolic and organelle-resident proteins, as was suggested for proteins known to be essential for plastid functions [40]. In contrast, the fluorescence of the stromal marker PTCK-CFP [42] overlapped with the autofluorescence of chlorophyll (Chl) (Figure 1b and Figure S2b). In agreement with Figure S1b, the ABCB28-GFP signal was also detected in plastids from the main and lateral roots from all cell layers, as was the PTCK-CFP signal (Figure 1c and Figure S2c).

### 2.2. IAA Transport across Chloroplast Envelopes Is Mediated by ABCB28 and Possibly by ABCB29

**Figure 2:**
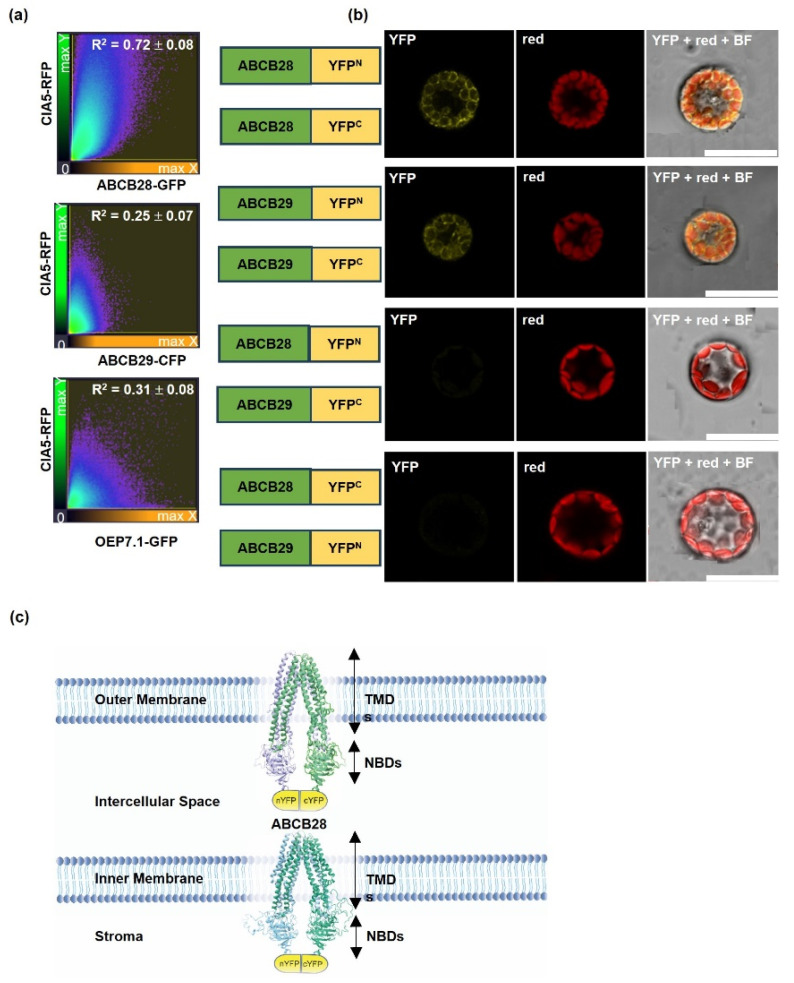
ABCB28 in the inner and ABCB29 in the outer chloroplast envelopes form homodimers. (**a**) Transient co-expression assays in *Arabidopsis* mesophyll protoplasts. Representative scatterplots show means ± SD Pearson’s correlation coefficient (R^2^, mean ± SD, n = 15 images, four protoplasts) at chloroplast envelope between ABCB28-GFP, ABCB29-CFP, and chloroplast outer envelope marker OEP7-GFP with the co-expressed CIA5-RFP chloroplast inner envelope marker, as indicated. (**b**) Bimolecular fluorescence complementation (BiFC) analysis using an *Arabidopsis* protoplast transient expression system and confocal microscopy analysis. Co-expression of ABCB28-nYFP and ABCB28-cYFP or of ABCB29-nYFP and ABCB29-cYFP resulted in a significant fluorescence signal in chloroplast envelope. Co-expression of ABCB28-nYFP and ABCB29-cYFP, and ABCB28-cYFP and ABCB29-nYFP were used as negative controls. Images were taken 16 h after transfection. Scale bars: 20 µm. Chlorophyll autofluorescence is shown as red. BF, bright field. Presented are representative images from three experiments (n = 9). Schematic representation of the constructs used are shown. (**c**) Predicted protein models of ABCB28 and ABCB29 homodimers built using SWISS-MODEL homology modeling server (https://swissmodel.expasy.org). Ribbon representation of the ABCB28 and ABCB29 homodimers with the two subunits, respectively, colored in violet and green for ABCB28 and in light blue and green for ABCB29. The C-terminal nucleotide binding domains (NBDs) of the ABCB28 and ABCB29 monomers are facing the stroma and the intermembrane space, respectively. The SWISS-MODEL template library was searched using BLAST and HHBlits for evolutionarily related structures matching the target sequence of the human mitochondrial ABCB10 (PDB code: 4ayx). The fluorescent protein tags fused to the NBDs of the ABCBs monomers used in BiFC assays are represented in yellow. Transmembrane domains (TMDs).

To further investigate the subplastidial localization of ABCB28 and ABCB29, we next conducted transient co-expression assays in *Arabidopsis* mesophyll protoplasts expressing *35S_pro_:ABCB28:GFP* or *35S_pro_:ABCB29:CFP* constructs co-transfected with the IE marker construct *CIA5_pro_:TM2:RFP* (CIA5-RFP) [45]. As a negative control, we co-transformed the protoplasts with *CIA5_pro_:TM2:RFP* and the OE marker construct *35S_pro_:OEP7:GFP* (OEP7-GFP) [46] (Figure S4). In agreement with a previous report showing ABCB28 in the IE [25], the fluorescence of ABCB28-GFP overlapped perfectly with that of CIA5-RFP. However, the ABCB29-CFP and CIA5-RFP signals did not co-localize (Figure 2a). To investigate whether ABCB28 and ABCB29 form homodimers, respectively, we performed bimolecular fluorescence complementation (BiFC) analysis on the *Arabidopsis* mesophyll protoplasts. The co-expression of ABCB28 fused at the C-terminus with the N-terminus of YFP (ABCB28-nYFP) and ABCB28 fused at the C-terminus with the C-terminus of YFP (ABCB28-cYFP), or ABCB29-nYFP and ABCB29-cYFP, resulted in a significant fluorescence signal in the chloroplast envelope (Figure 2b). These data strongly indicate that ABCB28 and ABCB29 homodimerize in vivo. On the contrary, no fluorescence signal was observed in protoplast cells co-expressing ABCB28-nYFP and ABCB29-cYFP or ABCB28-cYFP and ABCB29-nYFP (Figure 2b). In support of this, structural modeling using the SWISS-MODEL protein modeling server and derived from high-resolution crystal structures of the human mitochondrial ABCB10 [47] predicted ABCB28 and ABCB29′s quaternary structures as homodimers (Figure 2c). We predict the directionality of ABCB28 and ABCB29 transport based on the structures and topology studies of yeast, human, and *Arabidopsis* mitochondrial ABCB transporters (Figure 2c) [31,32,48,49].

**Figure 3:**
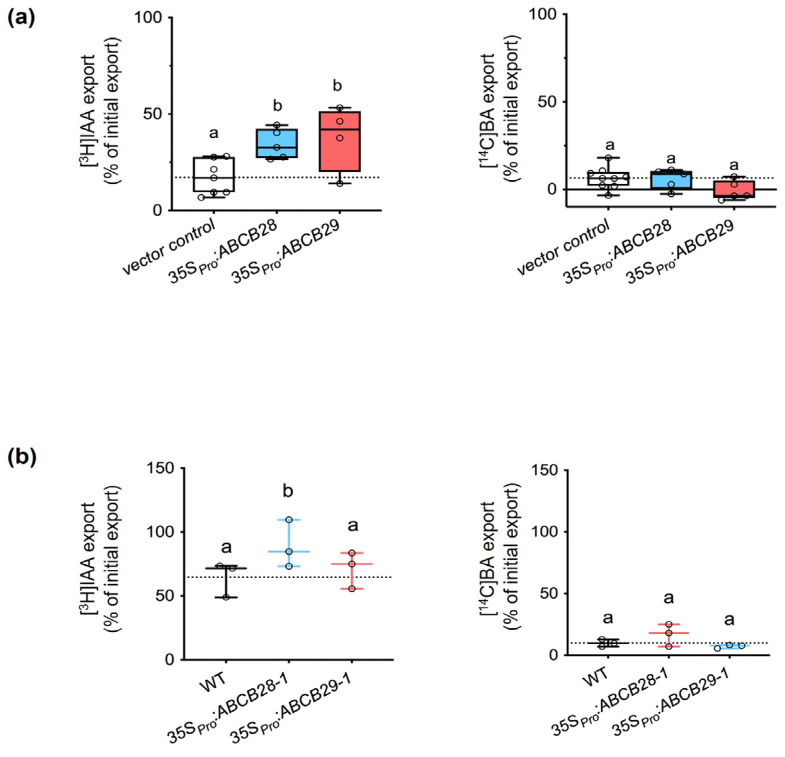
ABCB28 and ABCB29 catalyze auxin efflux from chloroplasts. (**a**) [^3^H]-IAA efflux from protoplasts prepared from tobacco leaves transfected with *35S_pro_:ABCB28*, *35S_pro_:ABCB29*, or empty vector (control). IAA export was significantly enhanced in protoplasts expressing *ABCB28* and *ABCB29* compared with vector control, while efflux of [^14^C]-BA (benzoic acid) did not differ significantly. Significant differences (*p* < 0.05) of means ± SE (n = four individual transfections and protoplast preparations) were determined using ordinary one-way ANOVA and are indicated by different lowercase letters. (**b**) [^3^H]-IAA and [^14^C]-BA efflux from chloroplasts prepared from wild-type (WT), *35S_pro_:ABCB28-1* and *35S_pro_:ABCB28-1 Arabidopsis* lines. Enhanced IAA export caused by constitutive overexpression of ABCB28 demonstrates the identity of ABCB28 as an inner envelope IAA transporter. Significant differences (*p* < 0.05) of means ± SE (n = four individual transfections and chloroplast preparations) were determined using ordinary one-way ANOVA and are indicated by different lowercase letters.

We next assessed whether ABCB28 and ABCB29 are involved in auxin transport. To avoid a negative effect of the fluorescence tags on the ABCB activities, we first quantified the IAA efflux from tobacco leaf mesophyll protoplasts prepared from leaves transfected with *35S_pro_:ABCB28* or *35S_pro_:ABCB29* constructs. The expression of both constructs greatly enhanced IAA export activity compared to the vector control (Figure 3a). [^3^H]-IAA export was specific, whilethe commonly used aromatic acid diffusion control benzoic acid (BA) was not differently exported from the *35S_pro_:ABCB28* and *35S_pro_:ABCB29* protoplasts (Figure 3a). Next, the IAA transport activities were directly investigated biochemically in isolated chloroplasts; the intactness of the chloroplasts was assessed using phase contrast light microscopy before and after transport [50] and found to be around 80–90% (not shown). Isolated chloroplasts from the WT and the stable transformant *35S_pro_:ABCB28-1* and *35S_pro_:ABCB29-1* plants (Figure S5) were loaded with radiolabeled [^3^H]-IAA followed by the immediate quantification of IAA export. The results clearly indicate that the overexpression of ABCB28 enhances [^3^H]-IAA but not [^14^C]-BA export out of the chloroplasts into the cytosol (Figure 3b). The fact that IAA efflux is not affected in the chloroplasts of the *35S_pro_:ABCB29-1* line could indicate that ABCB28 activity on the IE is the limiting step for auxin chloroplast efflux.

### 2.3. Arabidopsis Chloroplasts Actively Biosynthesize Auxin

**Figure 4:**
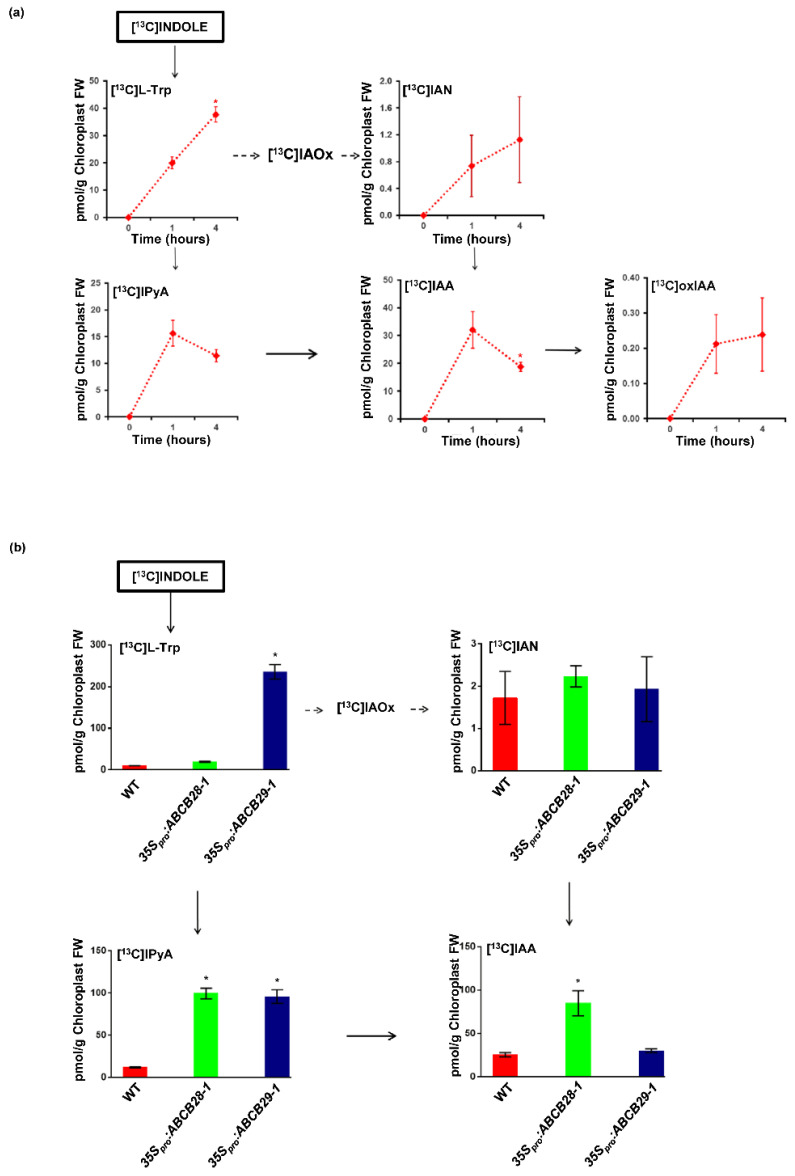
Biosynthesis of auxin in *Arabidopsis* chloroplasts. (**a**) Formation of labeled IAA, IAA precursors, and catabolites after incubation of isolated chloroplasts from 4-week-old *Arabidopsis* plants with [^13^C]-indole for 0, 1, and 4 h. Asterisks mark means that are significantly different after 1 h versus 4 h of incubation based on Student’s *t*-test (*p* < 0.01). (**b**) Biosynthesis of labeled IAA and IAA precursors after 1 h incubation of isolated chloroplast from 4-week-old *Arabidopsis* WT and *ABCB28,29* overexpressing plants determined using [^13^C]-indole. Asterisks mark means that are significantly different from those of WT chloroplasts based on Student’s *t*-test (*p* < 0.01). In (**a**,**b**), error bars represent SD of means. Samples were analyzed in four independent biological replicates. The concentrations for all metabolites are given in picomoles per gram of chloroplast FW. L-Trp, L-tryptophan; IPyA, indole-3-pyruvic acid; IAN, indole-3-acetonitrile; IAOx, indole-3-acetaldoxime; oxIAA, 2-oxindole-3-acetic acid.

Given that ABCB28 and ABCB29 act as IAA efflux carriers, we therefore tested whether *Arabidopsis* chloroplasts biosynthesize IAA. For this purpose, we performed feeding experiments using isolated chloroplast incubated with [^13^C]indole for 1 and 4 h. Liquid chromatography–tandem mass spectrometry (LC–MS/MS) analysis detected a rapid incorporation of [^13^C]indole into the IAA precursors, including tryptophan (Trp), indole-3-acetonitrile (IAN), and indole-3-pyruvic acid (IPyA). Incubation of 1 h exhibited higher labeling of IAA. The major IAA catabolite 2-oxindole-3-acetic acid (oxIAA) [51] was also detected (Figure 4a). The levels of other IAA precursors such as indole-3-acetaldoxime (IAOx), as well as of the IAA conjugates IAA-aspartate, IAA-glutamate, IAA-glucose (IAA-Glc), and oxIAA-Glc, were below the limit of detection. The results are consistent with a de novo IAA synthesis pathway in *Arabidopsis* chloroplasts.

Furthermore, we performed feeding experiments using *ABCB28*- and *ABCB29*-overexpressing lines to test whether greater IAA efflux activity affects chloroplast IAA metabolism. After one hour of incubation with [^13^C]indole, higher labeling of IPyA and IAA in the isolated chloroplasts from *35S_pro_:ABCB28-1* and of L-Trp and IPyA in the *35S_pro_:ABCB29-1* chloroplasts was detected vis à vis that seen in the WT plants. The rates of de novo synthesis of the intermediate IAN were, however, similar in all the lines (Figure 4b).

### 2.4. Spatial Expression Patterns of ABCB28 and ABCB29

To address this issue, we generated the *ABCB28_pro_:GUS* and *ABCB29_pro_:GUS* promoter reporter gene fusion constructs (Figure S6). The entire intergenic region of the ABCB28 and ABCB29 genes was used (see Materials and Methods). Under unstressed conditions, the GUS expression under *ABCB28* promoter activity was visible in the hypocotyl–root junction and in the main root tip and roots tips of mature lateral roots, but not in the lateral root primordia. The *ABCB29* promoter activity was most prominent on the margins of the petioles and at the bases of young leaf primordia. In the roots, weak GUS staining was restricted to the maturation zone of the primary root. Osmotic stress imposed using NaCl or PEG [3] induced GUS activity in the shoot–root junctions of the *ABCB28_pro_:GUS* seedlings, even as a slight decrease was observed in the root and lateral root tips. The GUS activity in the *ABCB29_pro_:GUS* seedlings was not affected, however, by the stress treatments. The discrepancy between the ABCB28 and ABCB29 gene (Figure S6) and protein (Figure 1 and Figure S2) expression patterns suggests that both processes are spatially unlinked. Protein-coding messenger RNAs can move between various organs in plants, suggesting that a postulated tissue-specific gene expression profile might not be predictive for the actual plant body part in which a transcript exerts its function [52].

### 2.5. Overexpression of ABCB28 and ABCB29 Alters the Response to Exogenously Applied Auxin

Auxin plays a role in root development. Excess amounts of IAA are known to promote secondary root initiation [53]. We studied this response in 7-day-old seedlings (not yet developed emerged lateral roots) that were transferred for 3 additional days into externally applied 0.1 μM IAA. Without IAA treatment, the number of emerged lateral roots was similar in all the lines (Figure S6a). However, transgenic lines were more sensitive to the IAA induction of lateral root growth (Figure S7a). The increased induction of lateral roots growth in the overexpressors was not related to a higher number of root primordia prior to the exposure to auxin, because the number of root primordia from stages I–VII [54] over the main root length of the 7-day-old seedlings was similar in all the lines (Figure S7b).

### 2.6. Overexpression of ABCB28 and ABCB29 Influences Stomatal Conductance

**Figure 5:**
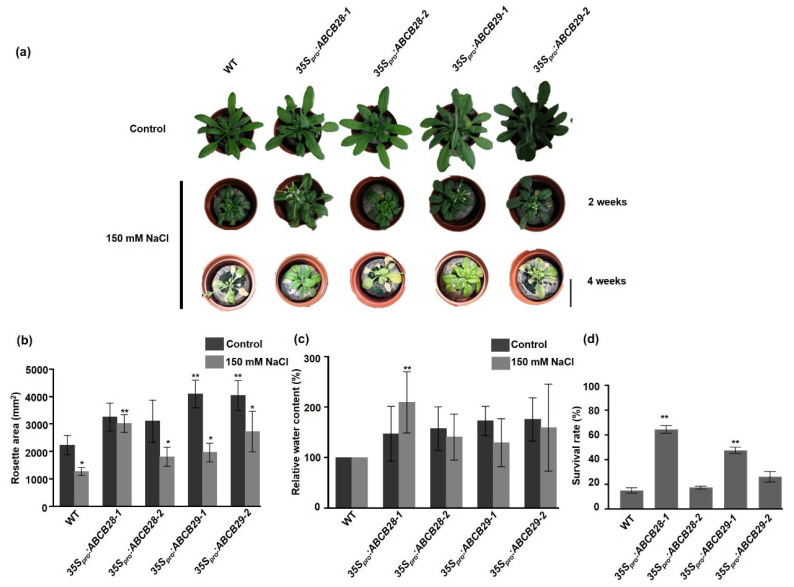
Increased tolerance of *35S_pro_:ABCB28* and *35S_pro_:ABCB29* plants to salt stress. (**a**) Rosette shapes of 5-week-old non-stressed (**top**) wild-type (WT) and *ABCB28/29* overexpressors growing under long-day conditions. Other plants were grown for 3 weeks and watered with a NaCl solution for 2 (**medium**) or 4 additional weeks (**bottom**) Scale bar, 9 cm. (**b**) Rosette area determined in 4-week-old plants which were non-treated or watered for 7 days with NaCl (mean ± SD, n = 10 from at least 3 independent experiments). (**c**) Relative shoot water content after 7 days of stress was not affected in *35S_pro_:ABCB28-1* plants (mean ± SD, n = 10 from at least three independent experiments). (**d**) Survival was assessed 4 weeks after stress and determined as the number of visibly green plants (n = 24 from at least three independent experiments). (**a**–**d**) One asterisk mark means that are significantly different from control conditions; two asterisks marks means that are significantly different from WT based on Student’s *t*-test (*p* < 0.01).

To explore the effects of altered levels of ABCB28 and ABCB29 on the plant phenotype, the WT and *ABCB28*- and *ABCB29*-overexpressing seedlings were cultured in soil. Despite exhaustive searches, we could not recover homozygous null *ABCB28* mutants. The fact that ABCB28 loss-of-function lines could not be identified suggests that *ABCB28* may be required for the plant’s survival. No phenotypic differences were observed between the WT and overexpressing plants (Figure 5a), except that *35S_pro_:ABCB29-1* and *35S_pro_:ABCB29-2* showed a slightly increased rosette size (Figure 5b). The bolting time (21 days), shoot water content (Figure 5c), levels of photosynthetic pigments, and Chla:b ratios (Table S1) were similar in all the lines. Moreover, no significant difference among lines were observed in the Normalized Difference Vegetation Index (0.85 ± 8 × 10^−3^), which estimates leaf Chl as well as nitrogen status [55]. We next assessed whether they exhibited altered photosynthesis. Chla fluorescence measurements also failed to reveal major changes in the maximum photochemical efficiency of photosystem II (PSII) in the dark-adapted state (Φ_PSIImax_), and in the photochemical efficiency of PSII (Φ_PSII_), photochemical quenching (*qP*), and non-photochemical quenching (*NPQ*) at different photosynthetically active radiation (PAR) levels (Figure S8a). Fast kinetics of fluorescence induction were recorded in the expanded leaves of the WT, *35S_pro_:ABCB28-1*, and *35S_pro_:ABCB29-1*, and various energy fluxes were calculated and expressed per PSII reaction center (RC). The extracted parameters are briefly described in Table S2 [56]. In line with the absence of a significant difference in the Chla:b ratio (Table S1), all the studied lines showed similar ABS/RC values (Figure S8b). Furthermore, we found no statistically significant differences between genotypes in their PSII physiological states as determined by TR0/RC and DI0/RC. Moreover, the maximum quantum yield of PSII (Phi_P0), quantum yield of electron transport (Phi_E0), probability that a trapped exciton enters the electron transport chain (Psi_0), and quantum yield of energy dissipation (Phi_D0) were similar in all the genotypes (Figure S8b). Taken together, these data indicate a similar photochemical efficiency and distribution of energy fluxes at the level of PSII in all the lines. Furthermore, all lines sustained similar CO_2_ assimilation rates (A, μmol m^−2^ s^−1^) at the PAR levels of the growth chamber (150 ± 50 μmol photons m^–2^ s^−1^) and at the WT plants’ light saturation point (500 ± 100 μmol photons m^–2^ s^−1^). Consistently, a similar intercellular CO_2_ concentration (Ci, ppm) was obtained in the WT and overexpressing plants (Table 1). However, at the light saturation point, the *35S_pro_:ABCB28-1* and *35S_pro_:ABCB29-1* plants displayed lower stomatal conductance of water vapor (GH_2_O, mmol m^–2^ s^−1^) (Table 1). Stomatal conductance refers to the capacity of the stomata to lose water [57]. Likewise, the transpiration rates (E, mmol m^–2^ s^−1^) and water use efficiency (WUE) were lower and higher, respectively, in the overexpressors than in the WT at 500 μmol photons m^–2^ s^−1^ (Table 1), thus indicating that transgenic lines are able to assimilate greater amounts of CO_2_ for a given amount of water at high light intensities. Thus, the results show that plastids are minor sources of cellular auxin under normal growth conditions, and that the overaccumulation of ABCB28 and ABCB29 in the chloroplast envelope of the guard cells (Figure 1a and Figure S2a) plays a role in stomata aperture under photoinhibition imposed by high light.

### 2.7. ABCB28 and ABCB29 Are Involved in Osmotic-Stress-Adaptive Response

The observed role of ABCB28 and ABCB29 on the WUE (Table 1) led us to assess the potential impact of their overexpression on salinity stress tolerance. After 2 weeks of NaCl stress, the *35S_pro_:ABCB28-1* plants showed a decreased stress-induced rosette compaction response and, like the *35S_pro_:ABCB29-1* line, had inflorescences with opened flowers, as well as a main stem longer than in the WT plants (Figure 5a,b). Consistent with an improvement in shoot growth, the total rosette area and relative shoot water content after 7 days of stress were not affected in the *35S_pro_:ABCB28-1* plants (Figure 5b,c). The levels of photosynthetic pigments and Chla:b ratios were similar in all the lines (Table S1). After 4 weeks of stress, the survival rates were comparable in the *35S_pro_:ABCB28-2* and WT plants. However, *35S_pro_:ABCB28-1*, *35S_pro_:ABCB29-1*, and *35S_pro_:ABCB29-2* showed increased survival rates of 66%, 46%, and 29%, respectively (Figure 5d). The severe foliar chlorosis and necrosis in the surviving plants is shown in Figure 5a. These results indicate that the tolerant phenotypes were attributable to the level of expression of the transgene and not to the genome position, where constructs were integrated after transformation. Consistent with the survival rate, the *NPQ* values of the *35S_pro_:ABCB28-1* and *35S_pro_:ABCB29-1* plants 7 days after treatment were slightly but significantly lower than those of the WT (Figure S8a), indicating a lower susceptibility to photoinhibition upon stress in the tolerant lines [58]. The fast kinetics of the fluorescence induction measurements also failed to show differences among lines after 7 days of NaCl stress (Figure S8b). Taken together, these results indicate that the transport activities of ABCB28 and ABCB29 influence the photosynthesis-protective excitation energy dissipation mechanisms. Gas exchange analysis evidenced a slight but significantly increased CO_2_ assimilation rate in the *35S_pro_:ABCB28-1* plants in comparison to the WT at the growing PAR level and at the saturating PAR level. The GH_2_O and WUE were also significantly decreased and increased, respectively, under growing and saturating PAR levels in both the *35S_pro_:ABCB28-1* and *35S_pro_:ABCB29-1* plants. These differences were most pronounced in *35S_pro_:ABCB28-1*. Lower E compared to the WT plants in growing PAR was also observed in both overexpressing lines, but CO_2_ flux from the air to the substomatal cavity was not affected, since the leaves of the WT and overexpressing plants showed similar Ci values (Table 1). In sum, these data indicate that the overexpression of ABCB28 and ABCB29 improves the WUE under NaCl stress via a reduction in E, substantiated by lower GH_2_O.

**Figure 6:**
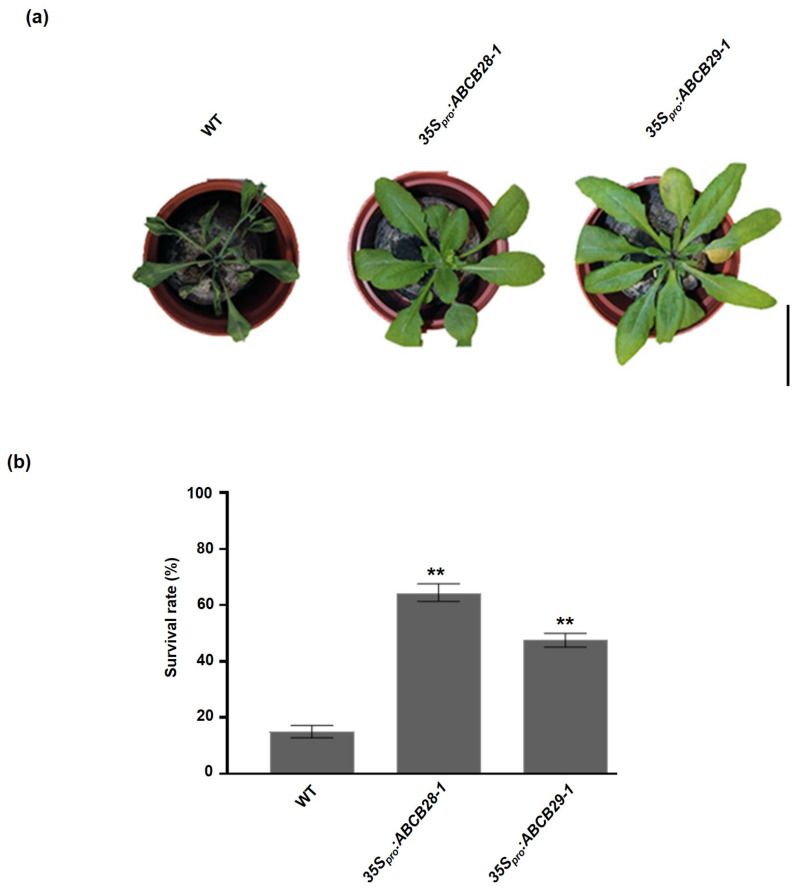
Overexpression of *ABCB28* and *ABCB29* improves water stress tolerance. (**a**) Plants were grown under a controlled watering regime (well watered) for 3 weeks, then deprived of further watering for 7 days and photographed. Scale bar, 1 cm. (**b**) On day 8, plants were re-watered and observed for recovery on day 12 (n = 20, at least three independent experiments). ** Significant differences from WT, based on Student’s *t*-test (*p* < 0.01).

The results led us to test the drought tolerance response of the transgenic lines. Plants were first grown for 3 weeks under water-sufficient conditions. Watering was then stopped altogether as a drought treatment. After 7 days, all WT plants looked severely dehydrated. By contrast, the *35S_pro_:ABCB28-1* and *35S_pro_:ABCB29-1* plants still looked healthy (Figure 6a). On day 8, all plants were re-watered. After 4 days of re-watering, 66% of the *35S_pro_:ABCB28-1* and 46% of the *35S_pro_:ABCB29-1* plants had regained turgor, while just 20% of the WT plants presented signs of recuperation (Figure 6b). Taken together, our observations clearly show that ABCB28 and ABCB29 contribute to water- and salinity-stress-adaptive responses.

## 3. Discussion

It is now clear that auxin transporting proteins are essential for the proper integration of environmental inputs into auxin signaling [1]. Here, we uncovered auxin transporters located in the chloroplast that are involved in salt and drought stress adaptation (Figure 1, Figure 2, Figure 3, Figure 5 and Figure 6). ABCB28 localizes in the IE and ABCB29 in the OE, where they seem to form homodimers, respectively (Figure 2 and Figure S4). This finding, to our knowledge, is the first IAA transport system to be identified thus far in chloroplasts. The enrichment of ABCB subfamily members in land plants [59] may have adaptive significance inasmuch as, besides ABCB28 and ABCB29, other ABCBs act as mediators of environmental cues in vascular plants [59,60,61,62]. Our protoplast assays in heterologous tobacco suggested the joint action of ABCB28 and ABCB29 in moving IAA out of the chloroplasts (Figure 3a). However, direct measurement of the IAA export from isolated *Arabidopsis* chloroplasts (Figure 3b) indicated that only ABCB28-dependent IAA export represents a limiting step, which is consistent with its IE localization. This hypothesis is supported by the finding that *ABCB28* or *ABCB29* overexpression does not affect the plant morphology but allows transgenic plants to survive for prolonged periods under similar stresses of salinity and drought (Figure 5 and Figure 6). The increased stress performance of *ABCB29*-overexpressing plants might be due to the enhanced ABCB28 protein abundance, as *ABCB28* transcription during salt and osmotic stress is upregulated in the shoot–root junction (Figure S6). Interestingly, ABCB28 is localized also in root plastids (Figure 1c); hence, one or more other transporters in the OE of shoot and root plastids may be coupled to ABCB28 activity. In fact, the failure to identify null *ABCB28* lines underlines its vital role. Our finding highlights the evolutionary and developmental importance of chloroplasts for intracellular auxin transport.

Chloroplasts are known to provide Trp-derived precursors for IAA biosynthesis, and the chloroplasts of barley [14], sunflower [15], and Alaska pea [16] have been shown to actively biosynthesize auxin. Here, we present further proof using stable isotope-labeled indole feeding studies that *Arabidopsis* chloroplasts synthesize IAA (Figure 4a). Rapid labeling of the pool of the IAA precursors, IAA, and oxIAA was observed within 1 h after the feeding treatment began (Figure 4a), revealing that IAA is rapidly synthesized and turned over within these organelles. The oxIAA could be produced by ROS-dependent IAA oxidation, peroxidase activities [63], or unknown chloroplast dioxygenases, like the two recently discovered cytosolic DIOXYGENASES FOR AUXIN OXIDATION 1 and 2 [64,65]. It is important to mention that even though the feeding experiments were performed in isolated chloroplasts, a similar IAA metabolic capacity is expected also in other plastids, such as root plastids.

**Scheme 1:**
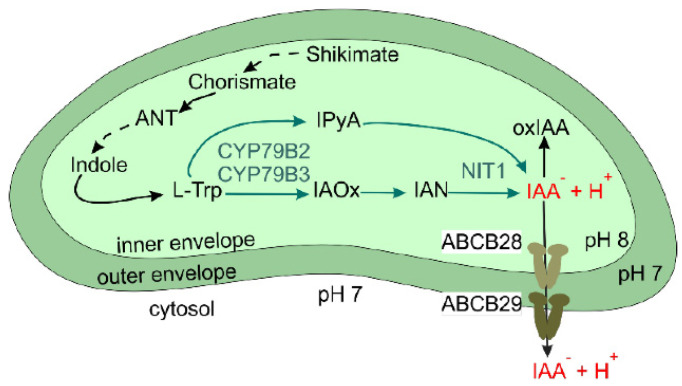
Hypothetical pathways of the IAA biosynthesis in chloroplasts. Black arrows indicate the biosynthesis of L-Trp in chloroplasts via the shikimate pathway. The L-Trp-dependent IAOx and IPyA auxin biosynthesis pathways are hypothesized to be present in chloroplasts and indicated by dark green arrows. IAOx could be channeled into biosynthesis of IAA via IAN and subsequent conversion of IAN into IAA catalyzed by NIT1. The enzymes known to operate in the IAOx pathway and predicted or confirmed to be targeted to plastids are shown in dark blue. At the alkaline pH of the stroma and envelope membrane space of chloroplast, the acidic proton of IAA is lost to yield anionic IAA^−^, which is unable to diffuse out of the chloroplast. The exit of IAA^−^ then needs to be assisted by active auxin anion efflux carriers at both the inner envelope and outer envelope membranes [80]. ANT, anthranilate (ATN); L-Trp, L-tryptophan; IPyA, indole-3-pyruvic acid; IAN, indole-3-acetonitrile; IAOx, indole-3-acetaldoxime, oxIAA, 2-oxindole-3-acetic acid; CYP79B2/3, cytochrome P450 monooxygenase 79B2/B3; NIT1, NITRILASE 1.

Three enzymes from the IAA indole-3-acetaldoxime (IAOx) biosynthesis pathway are predicted to be targeted to the chloroplast: two cytochrome P450s, CYP79B2 and CYP79B3 [6,66], and NITRILASE 1 (NIT1) [6,67]. NIT1–GFP fusions and proteomics analysis further confirmed NIT1 localization in both the plastids and cytoplasm [68,69,70,71]. The localization of these enzymes, on the one hand, and the accumulation of L-Trp, IAN, and IPyA in our feeding experiment, on the other, reveal the existence of an auxin IAOx-like [72] and IPyA-like [9,73,74,75] IAA biosynthesis pathway, respectively, in this organelle (Scheme 1). IAOx in the chloroplasts could be channeled into the biosynthesis of IAA via IAN and the subsequent conversion of IAN into IAA, catalyzed by NIT1 (Scheme 1). Analysis of *cyp79b2 cyp79b3*, *nit1-3*, and *NIT1* overexpressors revealed that the CYP79B2/3 and NIT1 pathways play a substantial role in the IAA levels in *Arabidopsis* plants [67,76]. The IAOx pathway is found only in *Brassicaceae* [6]. Therefore, most likely, not all terrestrial plants have conserved a chloroplast biosynthesis pathway during evolution. In plants, IAA is primarily synthesized via the IPyA pathway. TRYPTOPHAN AMIDOTRANSFERASE OF ARABIDOPSIS (TAA1) and TAA-related (TAR1) family proteins synthesize IPyA from Trp, which is then converted into IAA by the flavin monooxygenase (FMO) YUCCA family proteins [7,9,74]. In chloroplasts, some components of the chloroplast IAA metabolic pathways probably arose from pre-existing elements of the cyanobacterial ancestor. The Trp–IPyA-dependent production of IAA has been described in cyanobacteria. In this pathway, an aminotransferase is responsible for the conversion of Trp into IPyA, which is then converted into indole-3-acetaldehyde (IAAld) by indole-3-pyruvate decarboxylase (IPDC). IAA is then produced after the oxidation of IAAld by IAAld oxidase [77,78]. Speculation as to how Trp is converted into IPyA in the chloroplasts may include a pyridoxal 5′-phosphate-dependent D-amino acid aminotransferase with broad substrate specificity and acting on D-Trp as an amino donor [79]. In fact, in Alaska pea, IAA production was shown to occur in the plastids and involve a D-Trp aminotransferase that uses pyruvate as an amino group acceptor [16]. IPyA could then be converted into IAA, as occurs in cyanobacteria (Scheme 1). To obtain a more detailed characterization, however, will require further investigation.

*ABCB28* and *ABCB29* promoters are active at the sites of auxin synthesis and with the auxin concentration maxima (Figure S6), revealing the clear role of ABCB28 and ABCB29 in auxin distribution. The responsiveness of the *ABCB28* promoter under osmotic stresses (Figure S6) reflects the involvement of ABCB28 in determining the intracellular auxin levels and the consequent auxin-dependent stress responses. Thus, induction and repression of *ABCB28* transcription upon stress in the shoot–root junction and root tips, respectively (Figure S6), may be mechanisms by which environmental stress being sensed by the shoot is communicated to the roots with the aims of balancing auxin homeostasis and modulating growth and development accordingly. Moreover, increased ABCB28-dependent IAA efflux induces the de novo synthesis of IAA in the chloroplasts (Figure 4b). These results support the idea that plastid transporters, such as ABCB28, exert control over fluxes via metabolic networks by influencing the organellar metabolite concentrations [81]. For example, the downregulation of *Petunia hybrida* cationic amino acid transporter (PhpCAT), a plastid phenylalanine exporter, decreased in flowers the total cellular pools of phenylalanine and its plastids’ biosynthetic intermediates. Metabolic flux analysis revealed that flux via the plastid’s phenylalanine biosynthetic pathway is reduced in *PhpCAT* RNAi lines, indicating that the rate of phenylalanine export from the plastids contributes to regulating the flux via the aromatic amino acid network. On the contrary, elevated pools of phenylalanine and its intermediates were found in *PhpCAT*-overexpressing lines, which implies that higher efflux rates of phenylalanine induce the biosynthesis pathway in the plastids [81]. Another example of cross-regulation between transport and biosynthesis comes from studies conducted with *P. hybrid* plants overexpressing the plasma membrane strigolactone transporter PLEIOTROPIC DRUG RESISTANCE1 (PDR1), which belongs to the ABCG family and is localized in different root tissues. Increased strigolactone transport was accompanied by the induction of strigolactone biosynthesis gene expression in PDR1 overexpressors [82]. Both phenylalanine and strigolactone biosynthesis pathways are proposed to be feedback-inhibited by phenylalanine or strigolactone, respectively, and enhanced phenylalanine or strigolactone export from the site of their synthesis releases this feedback inhibition [81,82]. Our feeding assay monitored the rates of de novo synthesis of IAA but not its steady-state levels in the chloroplasts. Future metabolic flux analysis, coupled with IAA metabolite profiling using isolated chloroplast and leaves from transgenic plants, would further dissect the role ABCB28 plays in the plastid IAA biosynthesis network. In chloroplasts from *35S_pro_:ABCB29-1*, a similar effect on the IAA pathway seems to occur but with a delay in the conversion of IPyA into IAA (Figure 4b). The associated metabolic changes could be responsible in part for the better performance of the transgenic plants under stress. The importance of the chloroplast metabolism on the free IAA levels during stress adaptation was also evidenced in the *cyp79b2 cyp79b3* mutant, which had smaller rosette leaves than WT plants and showed increased severity in terms of shoot growth at high or low temperatures [76]. Taken together, these data suggest the substantial role of the chloroplast IAA biosynthesis pathway in resistance to environmental stress. Interestingly, the plastid redox NADPH thioredoxin reductase, NTRC, regulates auxin synthesis [83], raising the possibility that IAA biosynthesis in chloroplasts is under NTRC-dependent redox regulation. Further analysis of the impact of enhanced chloroplast IAA efflux under control conditions, salinity, and drought on the expression of cytosolic auxin synthesizing genes will give us hints as to how ABCB28 and ABCB29 are linked to the IAA biosynthesis pathways across these two subcellular compartments.

It is accepted that the transport of shoot-derived auxin from the shoot–root junction to the root tip is essential for the emergence, but not the initiation, of lateral roots [84,85,86]. The auxin response of *ABCB28*- and *ABCB29*-overexpressing plants in terms of lateral root growth (Figure S7a) further points to the role of chloroplast IAA in root growth and development but not in the de novo formation of lateral root primordia. Other examples of plastid proteins present in both green and non-photosynthetic tissues and affecting auxin-dependent root growth and development have been described [87,88]. For example, NTRC regulates auxin-dependent root and lateral root growth [87]. Interestingly, NTRC functions in the chloroplasts, but not in the amyloplasts, are necessary and sufficient to support WT rates of root growth and lateral root formation [87]. Moreover, increased levels of free IAA pools in *Arabidopsis* plants overexpressing *NIT1* lead to a strong reduction in the primary root length, accompanied by a higher number of shorter lateral roots and root hairs [67]. Crosses of knockout lines, such as of *cyp79b2 cyp79b3*, *nit1* [67], and *ntrc* [89], with *ABCB28* and *ABCB29* overexpressors and mutants could be of great scientific interest to shed light on the roles of plastids on auxin homeostasis.

Beside a homogeneous localization throughout the chloroplast envelope, ABCB28 and ABCB29 patches were observed when overexpressed as GFP fusion proteins (Figure S2a). Recent new data in the model alga *Chlamydomonas reinhardtii* show that most chloroplast envelope proteins have heterogeneous localization patterns, suggesting that they operate in specialized regions at the chloroplast envelope, acting as metabolic hubs that enhance or regulate specific reactions [90]. Knowledge on the role of these organizational features and of the molecular bases that underlie their organization is still sparce. Noteworthy is a recent work which sheds light on how clathrin-mediated endocytosis regulates chloroplast degradation during plant adaptation to environmental cues [91]. Upon abiotic stress, the protein CHLOROPLAST VESICULATION (CV) targets the chloroplast thylakoids and the IE. Envelope-membrane-localized CV leads to the formation of CV-containing vesicles (CVVs). A model was created in which damage or altered permeability of the OE induced by water or salt stress facilitated the interaction between CV and the cytosol-localized clathrin heavy chain. This interaction leads to vesicle budding from the chloroplast inner envelope toward the cytosol membrane. Via membrane scission, mediated by DYNAMIN-RELATED PROTEIN1A, the CVVs carrying the chloroplast proteins are subsequently released and mobilized to the vacuole for proteolysis [91]. In *Arabidopsis*, the overexpression of *CV* induced chloroplast degradation and premature leaf senescence, while silencing *CV* increased the chloroplast stability and prevented abiotic-stress-induced senescence [92]. Moreover, transgenic *CV*-silenced plants displayed enhanced tolerance to drought, salinity, and methyl-viologen-induced oxidative stress [92]. Interestingly, during water stress, the interaction of the oxidized cytoplasmic enzyme GLYCERALDEHYDE-3-PHOSPHATE DEHYDROGENASE (GAPC) with CV suppresses CV-induced chloroplast degradation [91]. GAPC is a key mediator in redox signal transduction in plants. Apart from its fundamental role in glycolysis, GAPC acts as a multifunctional protein in other biological processes, especially as a positive regulator of the response to abiotic stresses in plants [93,94,95]. Given the increased stress tolerance of the *35S_pro_:ABCB28* and *35S_pro_:ABCB29* plants (Figure 5 and Figure 6), an interesting question for future study is whether ABCB28 and ABCB29 mediate the plant response to salinity and water stress in part by suppressing CV’s function. It is tempting to speculate that the heterogeneous localization of ABCB28 and ABCB29 at the chloroplast envelope would facilitate the creation of niches of high IAA levels in the surrounding cytosol. Localized IAA-induced reactive oxygen species (ROS) production results in the perturbation of the cellular redox state [1] and could ultimately facilitate the oxidation of GAPC. We can speculate that confining the site of cytosolic ROS production to specialized auxin efflux sites at the chloroplast envelope could enhance GAPC–CV interaction. Therefore, maintenance of the chloroplast integrity under stress could be in part responsible for *35S_pro_:ABCB28* and *35S_pro_:ABCB29*′s increased stress tolerance (Figure 5 and Figure 6). The stable chloroplasts also could contribute to maintaining photorespiration, which is known to increase tolerance to abiotic stress by preventing photo-oxidative damage and optimizing photosynthesis [96,97]. In this regard, *CV* silencing increased water deficit stress tolerance in rice via the enhancement of nitrogen assimilation, CO_2_ fixation, and photorespiration [98]. Regarding the involvement of ABC transporters in ROS homeostasis, ABCG28 was shown to play a critical role in ROS accumulation at the tip of growing *Arabidopsis* pollen tubes. The sequestration of the ROS precursor polyamines into secretory vesicles is mediated by ABCG28 and helps the establishment of the tip-focused production of ROS, which is required for tip growth [99].

Promoting the WUE via a decrease in stomatal conductance can improve plant resistance to water-limiting stresses such as salinity and drought [100]. ABCB28 and ABCB29 in the guard cell chloroplasts (Figure 1a) are crucial for proper stomatal regulation at high light and in response to salinity (Table 1), suggesting the more general role of these transporters on stomata closure to protect the photosynthetic apparatus from photo-oxidative stress. Lower GH_2_O results in a higher WUE for conserving water (Table 1). This is closely associated with the increased photosynthetic CO_2_ assimilation and salt stress survival of the *ABCB28* and *ABCB29* overexpressors (Table 1, Figure 5). Lower *NPQ* values upon salt stress in overexpressing plants (Figure S8a) are consistent with an adaptive response [101]. It has been shown that an increased IAA concentration promotes stomatal closure [102]. Our results thereby indicate that chloroplast IAA synthesis and transport activity plays a role in stomatal behavior under photoinhibition conditions. Stomatal closure is a complex process that requires the integration of ROS and signaling molecules [103]. Enhanced chloroplast-derived IAA-dependent ROS production in the guard cells of *ABCB28* and *ABCB29* overexpressors could trigger stomatal closure. By utilizing auxin to regulate stomatal closure, chloroplasts may integrate photosynthesis and stomatal regulation in oxidative stress conditions. Examples of the chloroplast proteins involved in the ROS-dependent regulation of stomatal closure in response to environmental stresses include the proteins HCF106 and THF1. Both *thf1* and *hcf106* mutants displayed accumulation of ROS in the guard cells, increased stomatal closure, reduced water loss, and drought-resistant phenotypes compared to the WT [104].

Reduced E due to a reduction in GH_2_O may be caused by effects on the stomatal aperture or stomatal density. In general, a lower stomatal density is evidenced by a decline in GH_2_O in standard growth conditions [105,106,107]. Therefore, lower stomatal indices are unlikely to cause a higher WUE under high light and salt stress in *ABCB28-* and *ABCB29*-overexpressing plants, because no effect on GH_2_O was observed in control growth conditions (Table 1). For example, the reduction in stomatal density in *Arabidopsis* plants constitutively overexpressing the epidermal patterning factor EPF2 [107], loss-of-function *GT-2 LIKE 1* mutants [105], and *STOMAGEN* amiRNA mediated silencing lines [106] leads to a higher WUE and lower GH_2_O and E in standard growth conditions. Further studies on stomatal opening/closure, as well as the shape of guard cells, will provide a better understanding of how ABCB28 and ABCB29 contribute to the WUE under stress.

Drought and salinity are among the most important factors limiting plant performance and crop yields worldwide [108,109]. In this work, we provide a way to achieve an improved WUE and salt stress resistance in *Arabidopsis* with potential utility in crop biotechnology for improving agriculture in water-scarce and salt-affected areas of the world.

## 4. Materials and Methods

### 4.1. Plant Material and Growth Conditions

All *Arabidopsis* lines used were in the Col-0 ecotype. For the in vitro experiments, the seeds were germinated and grown on sterile plates containing 4.3 g l^−1^ Murashige and Skoog (MS) medium [110], 5 g l^−1^ sucrose, and 8 g l^−1^ agar in a controlled environment (22/18 °C, 60/80% humidity) and at a light intensity of 150 μmol m^–2^ s^–1^ radiation in a 16 h light/8 h dark photoperiod. The soil-grown plants were cultivated in growth chambers under similar conditions. For the preparation of the *Arabidopsis* mesophyll protoplasts, plants were grown under a 8 h light/16 h dark photoperiod.

### 4.2. Generation of Transgenic Lines

Full-length cDNAs of *ABCB28* (RAFL21-91-K22) and *ABCB29* (U10449) were used as the template for PCR reactions using Q5 High-Fidelity DNA Polymerase (New England Biolabs) and the primers ABCB28-F, ABCB28-R, ABCB29-F, and ABCB29-R, respectively (Table S3). To amplify the *ABCB28* and *ABCB29* coding regions without the stop codon, the reverse primers ABCB28-R2 and ABCB29-R2 were used instead (Table S3). The PCR products were cloned into pDONR207 (Invitrogen) and via recombination into the vectors pH7WG2 (*ABCB28* and *ABCB29*), pH7FWG2 (*ABCB28*), and pH7CWG2 (*ABCB29*) [111]. To study the expression patterns of the ABCB28 and ABCB29 genes, the entire upstream region from the *ABCB28* and *ABCB29* start codons to their respective neighboring gene (http://www.arabidopsis.org) were amplified using PCR. The amplified regions correspond to 1364 bp and 1040 bp, respectively. Col-0 genomic DNA, Q5 High-Fidelity DNA Polymerase, and the forward (ABCB28-PF, ABCB29-PF) and reverse (ABCB28-PR, ABCB29-PR) primers (Table S3) were used. The PCR products were cloned into pDONR207 and by recombination into PGWB633 [112] to generate the transcriptional *ABCB28_pro_:GUS* and *ABCB29_pro_:GUS* fusions. To generate the *ABCB28_pro_::ABCB28:GFP* and *ABCB29_pro_::ABCB29:GFP* constructs, the ABCB28 and ABCB29 promoter sequences and the ABCB28 and ABCB29 gene coding regions without the stop codons were amplified using PCR from genomic DNA with Q5 High-Fidelity DNA Polymerase using primers ABCB28-GF and ABCB28-GR for *ABCB28*, and ABCB29-GF and ABCB29-GR for *ABCB29* (Table S3). The PCR fragments were cloned into pDONR207 and by recombination into PGWB650 [112]. The *Arabidopsis* plants were transformed via the *Agrobacterium tumefaciens* GV3101/pMP90 strain-mediated floral dip [113]. Multiple transformants were selected using segregation analysis based on hygromycin and BASTA resistance. The overproduction of *ABCB28* and *ABCB29* in the *35S_pro_:ABCB* lines was confirmed using RT-PCR.

### 4.3. Reverse Transcription Polymerase Chain Reaction Analysis

The total RNA was isolated from the leaves using TRIzol reagent (Invitrogen), and the cDNA was synthesized from 5 μg total RNA with SuperScript IV RNase H-reverse transcriptase (Invitrogen) according to the manufacturer’s instructions. Each cDNA sample was diluted 1:10, of which 2 μL was used for PCR amplification with the AtABCB28-F and AtABCB28-R primers or AtABCB29-F and AtABCB29-R primers (Table S3). PCR amplification of the AtACTIN2 gene with the Actin-F and Actin-R primers (Table S3) was carried out as a loading control. The cycling parameters were 1 cycle at 94 °C for 2 min, followed by 30 cycles at 94 °C for 30 s, 50 °C for 30 s, and 72 °C for 2 min, plus a 5-min extension at 72 °C. The PCR products were detected using 2% agarose gels. All reactions were carried out in triplicate with two independent RNA samples.

### 4.4. Promoter GUS Analysis

Two-week-old seedlings grown on plates were immersed into MS or MS supplemented with 250 mM NaCl or 50 mM polyethylene glycol (PEG) 6000 solutions for 5 h. After GUS (β-glucuronidase) staining [114], the material was cleared in chloral hydrate solution (2.5 g of chloral hydrate in 1 mL of 30% glycerol). The samples were photographed using a Zeiss Axioscope upright microscope (Carl Zeiss AG, Munich, Germany)) or Olympus SZX16 microscope (Olympus, Tokyo, Japan) equipped with an Axiocam 105 color camera (Carl Zeiss AG, Munich, Germany).

### 4.5. Measurement of Rosette Area, Root Length, and Lateral Root Response

For the auxin-responsive root elongation and lateral root development assays, 7-day-old seedlings grown vertically on plates were transferred to new plates supplemented with IAA and grown under yellow-filtered light for an additional 3. Digital images of the seedlings were captured using a V700 scanner (Epson, Long Beach, CA, USA) and the root length quantified using the ImageJ software, V 1.8.0 (https://imagej.en.softonic.com/). For the lateral root assays, primordia emerging from the primary root, were counted as lateral roots. The numbers of root primordia were counted before the IAA treatments. The number of lateral roots and the number of root primordias were counted using an Axioscope 5 upright microscope (Carl Zeiss AG, Munich, Germany).

### 4.6. Measurement of Rosette Area

The rosette area was extracted from the FluorCAM FC 800-C imaging system (Photon Systems Instruments, Drásov, Czech Republic) data analysis. All assays were repeated a minimum of three times, with two replicates for each trial, and at least 15 seedlings per replicate were used.

### 4.7. Stress Treatments

Salt and drought stress tolerance was investigated using plants grown in separate pots in a controlled growth chamber for 3 weeks. The salinity was assayed by watering the plants with water containing 150 mM NaCl for 4 weeks, after which the survival rate was determined. The relative water content was obtained 7 days after treatment by calculating the difference between the fresh weight and dry weight of the shoots. Drought stress was imposed by withholding water for 7 days. After re-watering, plants which survived were counted.

### 4.8. Arabidopsis and Tobacco Protoplast Preparation

Intact *Arabidopsis* and tobacco (N. benthamiana) mesophyll protoplasts were prepared from the leaves of plant grown on soil under white light (100 µmol m^–2^ s^−1^), as previously described [115]. In short, the cell walls were digested by 1-3 h incubation at 30 °C in digestion buffer (0.3% pectolyase 1% cellulase, 1% BSA) in MCP (MCP: 500 mM sorbitol, 1 mM CaCl_2_, 10 mM MES, pH 5.6). The protoplasts were harvested via centrifugation after the addition of a 100% Percoll (500 mM sorbitol, 1 mM CaCl_2_, 20 mM MES, pH 6) cushion to the bottom of the tube. The protoplasts were collected and diluted in 100% Percoll, and intact protoplasts were isolated via centrifugation of the following gradient: protoplasts in 100% Percoll/1 vol. Percoll 30%/5 mL MCP. Intact protoplasts were collected from the Percoll 30%/MCP interphases. The tobacco protoplast preparation was identical to the *Arabidopsis* protocol except that a 25% Percoll gradient was used.

### 4.9. IAA Transport Assays in Isolated Tobacco Protoplasts and Arabidopsis Chloroplasts

The auxin transport experiments were performed as previously described [115]. In short, equal loading of intact protoplasts or chloroplasts (prepared by rupturing protoplasts by several syringe passages) with radiolabeled substrates was achieved via incubation with 1 µL ml^−1^ [^3^H]-IAA (specific activity of 20 Ci mmol^−1^; American Radiolabeled Chemicals, Inc., St. Louis, MO, USA) and 1µL ml^−1^ [^14^C]-benzoic acid (BA) (specific activity of 50 Ci mmol^−1^; American Radiolabeled Chemicals, Inc., St. Louis, MO, USA) on ice, excluding unwanted transport during the loading procedure. External radioactivity was removed using Percoll gradient centrifugation. The chloroplast intactness (showing a highly reflective, bright opaque appearance and the presence of a halo) was assessed prior and after transport using phase contrast light microscopy [50] and was around 80–90%. Efflux was started using incubation at 25 °C and halted using silicon oil centrifugation. The effluxed radioactivity was determined via scintillation counting of the aqueous phases and is presented as the relative efflux, which was calculated as follows: ((radioactivity in the medium at time t) − (radioactivity in the medium at time t = 0)) × (100%)/(radioactivity in the medium at t = 0). [^14^C]-BA served as a diffusion control of auxin transport.

### 4.10. Transient Expression with Particle Bombardment

Particle bombardment was carried out with the abaxial side of the *Arabidopsis* rosette leaves. Gold particles (1 mg, Bio-Rad Laboratories, Hercules, CA, USA) were coated with 2.5 to 5 μg plasmid DNA, 17 mM spermidine, and 1.25 mM CaCl_2_ and subsequently washed in 100% ethanol. Tissues were bombarded using the Biolistic^®^ PDS-1000/He particle delivery system (Bio-Rad Laboratories) using 1350 psi rupture disks at a distance of 60 mm and a vacuum of 85 kPa. The bombarded tissues were kept for 24 h in a dark humidity chamber at room temperature prior to microscopic analyses.

### 4.11. Confocal Microscopy and Image Acquisition

The confocal laser scanning microscopy work was performed using an LSM780 Airyscan (Inverted Axio, Carl Zeiss AG, Munich, Germany). The optics employed consisted of a C-Apochromat X40 water immersion lens (Carl Zeiss AG, Munich, Germany). The settings to record the emission of different fluorophores were: GFP and YFP (excitation 488 nm, emission 405–550 nm), CFP (excitation 458 nm, emission 473–495 nm), propidium iodide (excitation 495 nm, emission 635 nm), and Chl emission using a 620 nm long-pass filter (Carl Zeiss AG, Munich, Germany). All images were acquired with the pinhole diameter set to 1 Airy Unit. The leaf samples were mounted in perfluorodecalin onto slides [116].

### 4.12. Quantification of Colocalization

Z-stacks of the confocal images were uploaded into Imaris (Version 9.1.0, Bitplane) and the colocalization function was used to provide a quantitative measurement and statistical analysis. A total of 10 regions of interest were selected for each analyzed line and the fluorescence intensity profile of the corresponding color channels along the line was measured and plotted on the graph. A Pearson’s correlation coefficient was calculated using the software for each channel. Background correction values were automatically adjusted for all images.

### 4.13. Bimolecular Fluorescence Complementation (BiFC) and Transient Co-Expression Assays

The cDNAs without the stop codons of ABCB28 and ABCB29 were cloned into *pGWnY* (*nYFP*/*pUGW2*) and *pGWcY* (*cYFP*/*pUGW2*) BiFC expression vectors [117] to generate the *35S_pro_:ABCB28:nYFP*, *35S_pro_:ABCB28:cYFP*, *35S_pro_:ABCB29:nYFP*, and *35S_pro_:ABCB29:cYFP* plasmids. The nYFP and cYFP construct pairs were co-transformed into *Arabidopsis* protoplasts. The co-transformed protoplasts with *CIA5pro:TM2:RFP* and the OE marker *35S_pro_:OEP7:GFP* [46] in the colocalization experiments were used as negative controls. For the BiFC and colocalization experiments, intact *Arabidopsis* mesophyll protoplasts were isolated from 4-week-old plants, transfected, and cultured in darkness overnight at room temperature [118]. The fluorescence signals were visualized using CLSM Zeiss LSM780 (Carl Zeiss AG, Munich, Germany).

### 4.14. Gas Exchange and Chlorophyll a Fluorescence Parameter Measurement

For all the assays, the plants were adapted to darkness for 30 min before measurement. Gas exchange was carried out using an *Arabidopsis* whole-plant chamber and a Portable Gas Exchange Fluorescence System GFS-3000 (Heinz Walz GmbH, Effeltrich, Germany). The net CO_2_ assimilation (A), intercellular CO_2_ concentration (Ci), water vapor conductance (GH_2_O), and transpiration rates (E) were measured at a variable light intensity (0, 150 and 500 μmol m^–2^ s^–1^), a CO_2_ concentration of 400 ppm, and 21 °C. The measurement of Chl*a* fluorescence was performed on whole *Arabidopsis* rosettes using a FluorCam FC 800-C imaging system and analyzed with the FluorCam6 software package (ver. 1.8, Photon System Instruments, Drásov, Czech Republic). The darkness-level fluorescence yield (F_0_) was recorded using a 5 s flash of light (less than 1 μmol photon m^–2^ s^–1^). The maximum fluorescence yield in the dark-adapted state (F_m_) was measured at a saturating flash of about 8000 μmol photons m^–2^ s^–1^ for 800 ms. The plants were relaxed in darkness for 17 s and then subjected to 70 s of cool-white actinic lights. The coefficients photochemical quenching (*qP*), non-photochemical quenching (*NPQ*), and PSII quantum yield in light (Φ_PSII_) were measured at steady-state photosynthesis. The fast transient Chl*a* fluorescence was measured using the portable FluorPen FP 100 (Photon Systems Instruments, Drásov, Czech Republic) on the expanded leaves. Chl*a* fluorescence transients were induced by high irradiance (3000 µmol m^–2^ s^–1^), and fluorescence was recorded for 2 s.

### 4.15. Chlorophyll Content

The Chl in the leaves and chloroplasts was determined spectrophotometrically in acetone extracts [119]. The Normalized Difference Vegetation Index (NDVI) was recorded using a FluorCam PAR Absorptivity Module (Photon Systems Instruments, Drásov, Czech Republic).

### 4.16. Feeding Experiments and LC–MS Analysis of Auxins

Intact chloroplasts were isolated from the expanded leaves of 4-week-old plants using Percoll gradient centrifugation [120] and incubated in liquid medium containing 10 μM [^13^C]indole for 0, 1, and 4 h under gentle shaking. For each time point, chloroplasts from 10 g of leaves were collected in four replicates. The samples were extracted and purified using solid phase extraction as described in [121]. The chloroplasts were centrifuged and pellets were resuspended with 1 mL cold phosphate buffer (50 mM, pH 7.0) containing 0.1% sodium diethyldithiocarbamate, supplemented with internal standards. After centrifugation at 36,670 g for 10 min, one-half of each sample was acidified with 1 M HCl to pH 2.7, then purified using solid phase extraction (SPE) using Oasis^TM^ HLB columns (30 mg, 1 mL; Waters, Milford, CT, USA). For the quantification of IPyA, the second half of the sample was derivatized with cysteamine (0.25 M, pH 8.0) for 1 h, acidified with 3 M HCl to pH 2.7, and then purified using SPE. After evaporation under reduced pressure, the samples were analyzed using the Acquity UPLC I-Class (Waters, Milford, CT, USA) linked to a triple quadrupole mass spectrometer (Xevo TQ MS; Waters, Wrotham, UK). Incorporation of the labeled indole into the IAA precursors and metabolites was measured using LC–MS/MS, with the multiple-reaction monitoring transitions corresponding to the labeled compounds. The synthesis of the IAA precursors and metabolites was expressed as the concentration of labeled compounds after correction for natural isotope abundances.

### 4.17. Statistical Analysis

All numerical data were statistically analyzed using GraphPad PRISM V7.0 (GraphPad Software, San Diego, CA, USA). Student’s *t*-test were performed at a 95% confidence level and *p*-values determined. Data normality analyzed using one-way analysis of variance (ANOVA) were tested using Shapiro–Wilk test with a *p* = 0.05 rejection value prior to further analysis. Pairwise comparisons between treatments were performed when needed, using the appropriate post hoc tests, in our case, Sidak’s test. The type of analysis and *p* values are provided in the legends of figures and Supporting Information.

### 4.18. Accession Numbers

Sequence data from this article can be found in GenBank under the following accession numbers: *ABCG28* (AT4g25450), *ABCB29* (AT5g03910), and *RGS1-HXK1 INTERACTING PROTEIN 1* (AT4g26410).

## Figures and Tables

**Table 1:** Photosynthetic parameters under control and salt stress treatments.

**Control**						
	**PAR**	**A** **(mmol m^−2^ s^−1^)**	**GH_2_O** **(mmol m^−2^ s^−1^)**	**E** **(mmol m^−2^ s^−1^)**	**WUE**	**Ci (ppm)**
WT	150	2.61 ± 0.24	1194.30 ± 359.40	4.96 ± 2.24	0.80 ± 0.44	389.57 ± 3.71
	500	6.48 ± 0.80	748.07 ± 408.76	5.20 ± 3.14	1.75 ± 0.89	375.70 ± 0.33
*35S_pro_:ABCB28-1*	150	2.27 ± 0.18	819.50 ± 40.45	**1.89 ± 0.15**	1.21 ± 0.19	387.3 ± 1.86
	500	5.96 ± 0.63	**748.07 ± 19.61**	**1.99 ± 0.11**	**3.01 ± 0.47**	371.04 ± 0.89
*35S_pro_:ABCB29-1*	150	2.50 ± 0.07	1070.99 ± 302.83	2.75 ± 0.86	1.01 ± 0.32	387.71 ± 0.53
	500	6.19 ± 0.38	**806.60 ± 49.25**	**2.92 ± 0.30**	**2.92 ± 0.60**	372.80 ± 3.82
**150 mM NaCl**						
	**PAR**	**A** **(mmol m^−2^ s^−1^)**	**GH_2_O** **(mmol m^−2^ s^−1^)**	**E** **(mmol m^−2^ s^−1^)**	**WUE**	**Ci (ppm)**
WT	150	3.14 ± 0.10	1722.27 ± 251.77	7.97 ± 2.22	0.42 ± 0.10	387.81 ± 1.70
	500	5.33 ± 0.26	1667.33 ± 263.75	8.26 ± 2.52	0.70 ± 0.19	383.20 ± 1.90
*35S_pro_:ABCB28-1*	150	**3.77 ± 0.43**	**931.27 ± 191.48**	**3.82 ± 1.92**	**1.17 ± 0.44**	384.15 ± 1.27
	500	**7.88 ± 0.93**	**910.64 ± 171.39**	**3.82 ± 1.81**	**2.37 ± 0.76**	**371.40 ± 2.30**
*35S_pro_:ABCB29-1*	150	3.43 ± 0.13	**1374.07 ± 223.93**	**5.58 ± 2.02**	**0.72 ± 0.31**	386.46 ± 1.90
	500	6.30 ± 0.39	**1237.06 ± 258.51**	**5.67 ± 2.20**	**1.32 ± 0.58**	378.30 ± 2.20

Photosynthesis was measured at 150 µmol m^–2^ s^–1^ and 500 µmol m^–2^ s^–1^ as described in Materials and Methods. Well-watered WT and transgenic plants were grown in soil for 3 weeks under long-day conditions and then treated or not treated with NaCl for 1 week. Data are means ± SD. Bold values denote statistical significance (n = 3 from two independent experiments, Student’s *t*-test, *p* < 0.01). A, CO_2_ assimilation rate; GH_2_O, stomatal conductance; E, transpiration rate; WUE, water use efficiency; Ci, intercellular CO_2_ concentration.

## Data Availability

The data are contained within the article.

## Supplementary Materials

The following supporting information can be downloaded at https://www.mdpi.com/article/10.3390/plants13010007/s1, Figure S1: Amino acid sequence of transit peptides and gene expression analysis for ABCB28 and ABCB29. Figure S2: Transient transformation of *Arabidopsis* mesophyll cells. Figure S3: Expression pattern of ABCB28-GFP and ABCB29-CFP protein fusion in constitutive *Arabidopsis* transformants using confocal laser scanning microscopy. Figure S4: Subcellular localization of ABCB28-GFP and ABCB29-CFP fusion proteins in *Arabidopsis* leaf mesophyll protoplasts. Figure S5: Identification of homozygous *ABCB28*- and *ABCB29*-overexpressing lines. Figure S6: *ABCB28* and *ABCB29* are expressed in cells that produce auxin. Histochemical GUS staining of 2-week-old *ABCB28_pro_:GUS* and *ABCB29_pro_:GUS* seedlings under control conditions or exposed for 5 h to 250 mM NaCl or 50 mM polyethylene glycol (PEG). Figure S7: *ABCB28* and *ABCB29* overexpression in plastids alters auxin response. Figure S8: Altered photosynthetic capacity in *ABCB28-* and *ABCB29-* overexpressing plants. Table S1: Effect of salt stress on photosynthetic pigments. Table S2: Extracted and technical parameters from the OJIP protocol. Table S3: Primers used in this study.
