# Supplementary material for: Chloroplast Auxin Efflux Mediated by ABCB28 and ABCB29 Fine-Tunes Salt and Drought Stress Responses in Arabidopsis
Source: Plants (Basel). 2023 Dec 19;13(1):7. doi: 10.3390/plants13010007 (PMC10780339; doi:10.3390/plants13010007)
Supplement: Supplementary file 1 [file plants-13-00007-s001.zip › plants-2669021-supplementary.pdf]

Supplementary files

(A)

TP-ABCB28 MASATTLLFHHGSTRVLVARRRCQASVLRPYGGLKPFLSFCSLPNSTAPFRDSLRAKSDGLARAY-VT

TP-ABCB29 MSFLLLTPPPCLLIPPPPLSHRRSSSLFLKHPFQPSPRPLSFCKPSALRLRANTTVNS-LK

(B)

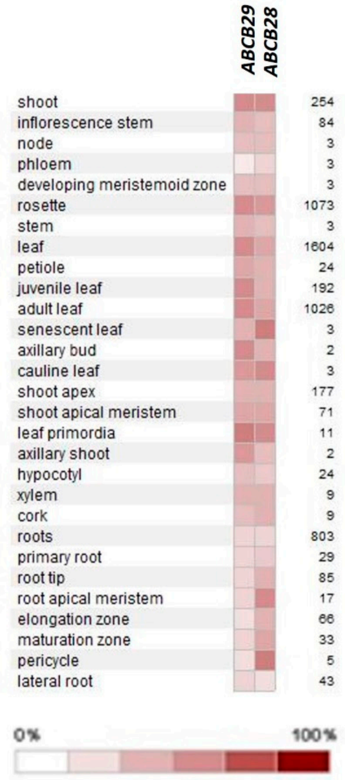

Figure S1. Amino acid sequence of transit peptides and gene expression analysis for ABCB28 and ABCB29. (A) ABCB28 and ABCB29 proteins have a transit peptide in amino acids 1-65 and 1-58, respectively. Red arrows indicate predicted processing cleavage sites. (B) Meta-profile heat map analysis of ABCB28 and ABCB29 genes expression in different anatomy parts during development. The microarray datasets used represent Genevestigator sets AT_AFFY_ATH1-0. Absolute values are rendered in pink-white, and the colours are normalised to the maximum value. The darkest colour corresponds to the maximum expression potential value for each gene.

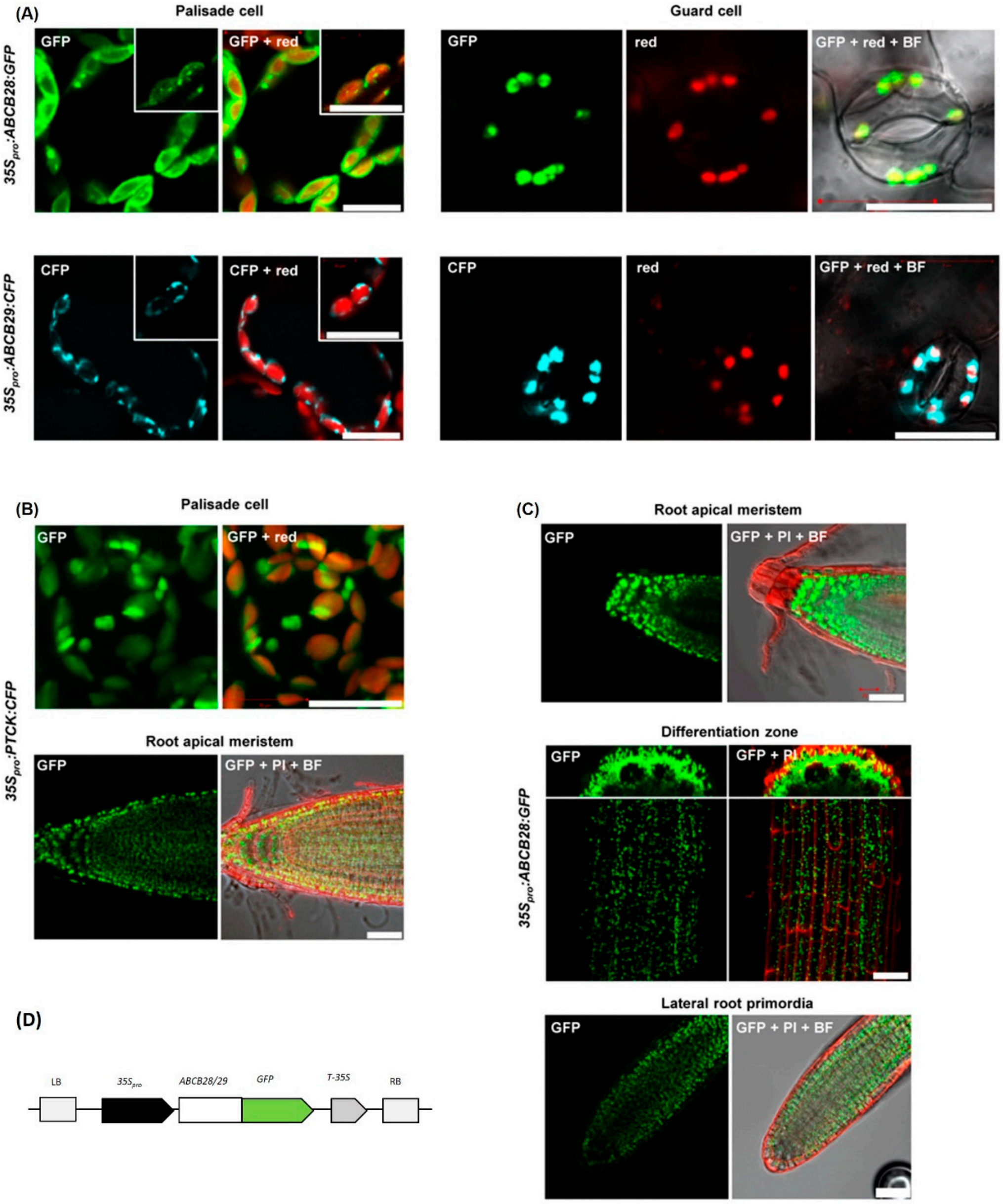

Figure S2. Expression pattern of ABCB28-GFP and ABCB29-CFP proteins fusion in constitutive *Arabidopsis* transformants by confocal laser scanning microscopy.

(A) Images reveal localization of both ABCB28-GFP and ABCB29-CFP fluorescence signals to chloroplast of palisade mesophyll cells and guard cells from leaves excised from *35S*$_{pro}$*:ABCB28:GFP* and *35S*$_{pro}$*:ABCB29:CFP* transgenic plants. Scale bars: 20 m. Insets show close-ups of chloroplasts in mesophyll cells. Scale bars: 5 m. (B) Confocal images of leaf palisade mesophyll and propidium iodide (PI) stained (red) root apical meristem cells from the stromal marker *35S*$_{pro}$*:PTCK:CFP* line. Scale bars: 20 m.

(C) ABCB28-GFP fluorescence signal is observed in plastids of cells from root apical meristem, differentiation root region, and lateral root primordia from *35S*$_{pro}$*:ABCB28:GFP* roots stained with PI. Scale bars: 20 m.

(A-C) Chlorophyll autofluorescence is shown as red. BF, bright field. Presented are representative images from two experiments and from seedlings 21-day-old.

(D) Schematic representation of the constructs used for the generation of transgenic lines. T-35S; 35S terminator and poly(A) signal.

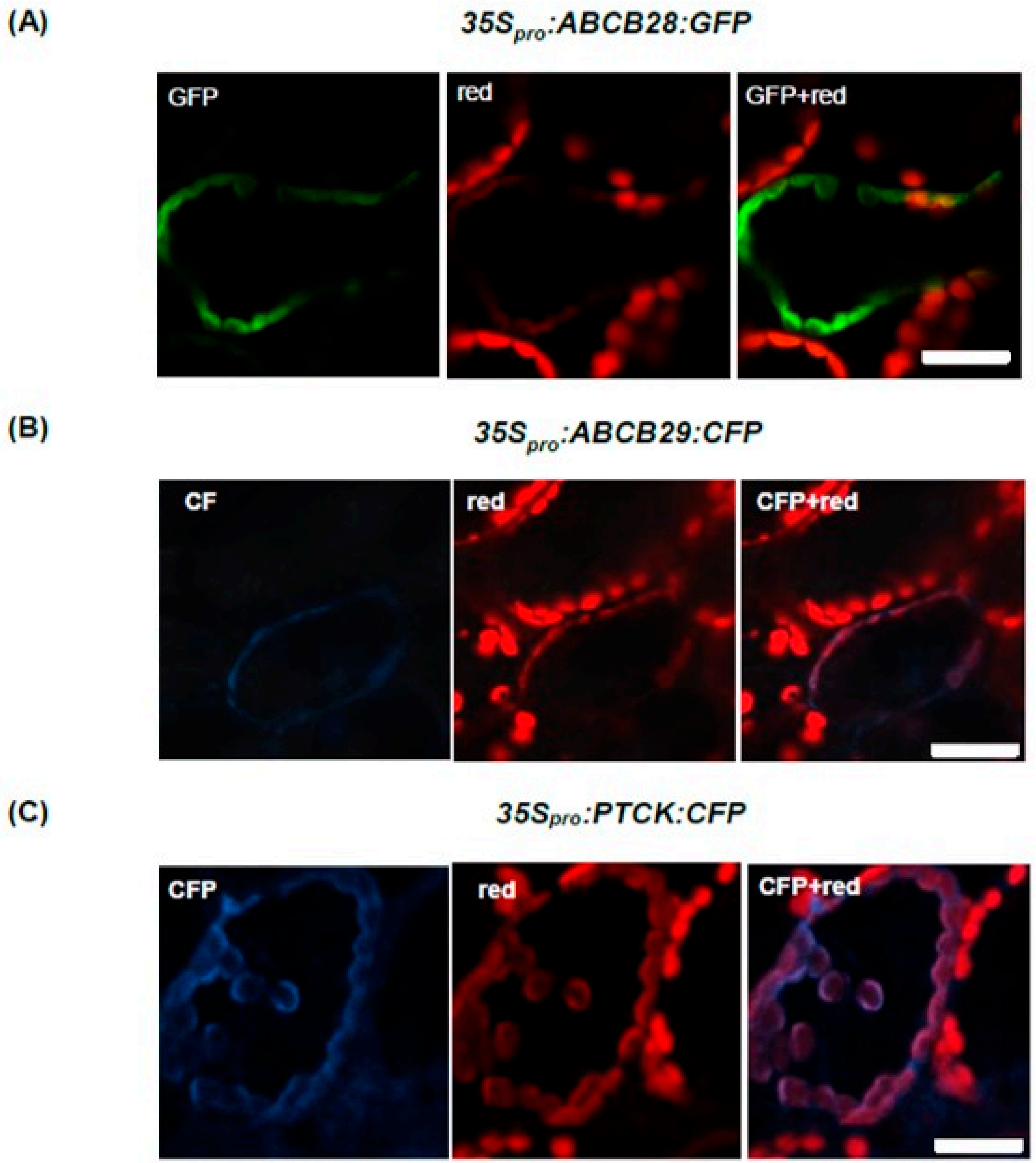

Figure S3. Transient transformation of *Arabidopsis* mesophyll cells. Plants were grown under short day for 4-5 weeks. (A) Plasmids harbouring $35S_{pro}$:ABCB28:GFP, (B) *35S$_{pro}$:ABCB29:CFP*, (C) and plastid marker *35S$_{pro}$:PTCK:CFP* constructs were bombarded into *Arabidopsis* leaves. After 24h, visualization by confocal laser scanning microscopy in mesophyll

was performed, Scale bars: 20 μm. Chlorophyll autofluorescence is shown as red.

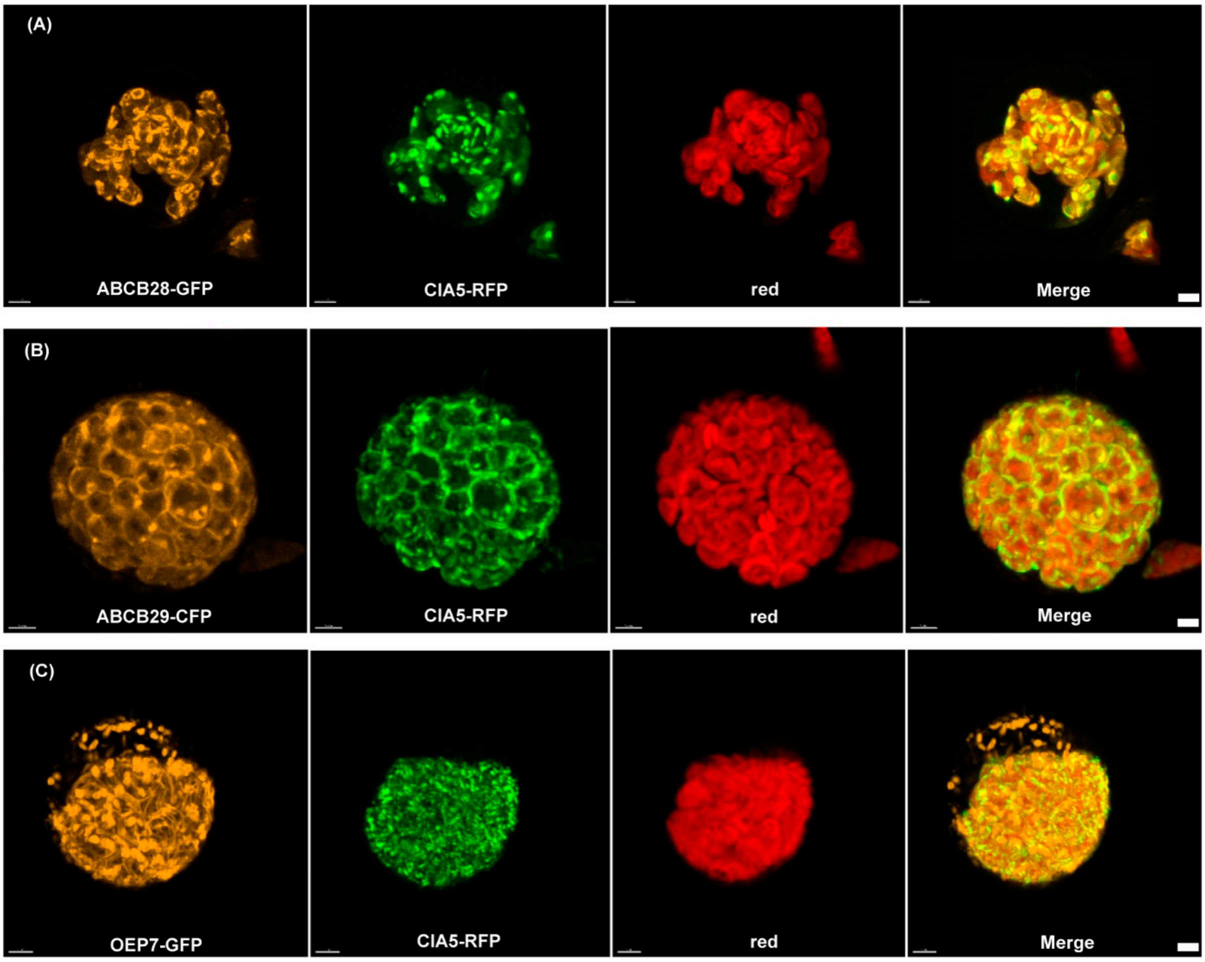

Figure S4. Subcellular localization of ABCB28-GFP and ABCB29-CFP fusion proteins in *Arabidopsis* leaf mesophyll protoplasts. Confocal laser scanning micrographs of a protoplast co-expressing ABCB28-GFP and the chloroplast inner membrane protein fusion CIA5-RFP (A), ABCB29-CFP and CIA5-RFP (B) and CIA5-RFP and chloroplast outer envelope marker OEP7-GFP (C). Results are representative of multiple cells in independent transfections. Chlorophyll autofluorescence (red). Images were generated after 16 hours of transfection. Scale bars = 5 μm.

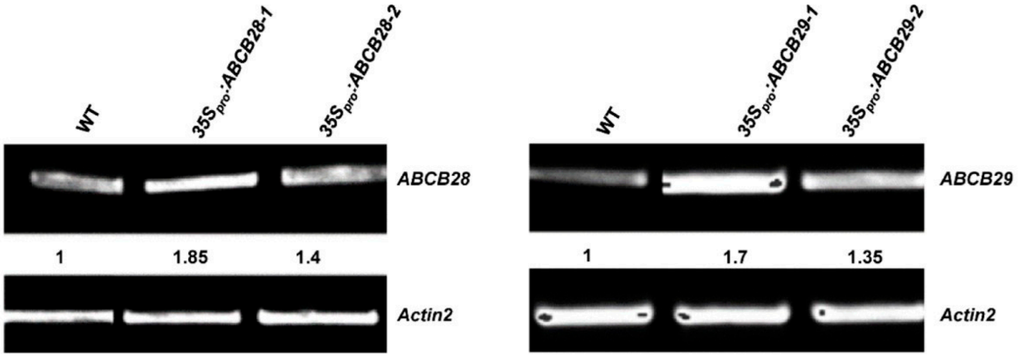

Figure S5. Identification of *ABCB28*- and *ABCB29*-overexpressing lines.

ABCB28 and *ABCB29* mRNA levels in wild-type (WT) and two *35S*$_{pro}$*:ABCB28* (*ABCB28-1*, *ABCB28-2*) and *35S*$_{pro}$*:ABCB29* (*ABCB29-1*, *ABCB29-2*) plants assessed by RT-PCR. The *Actin2* gene served as a control. Numbers below bands indicate relative abundance of *ABCB28* and *ABCB29* mRNA in the transgenic lines relative to WT.

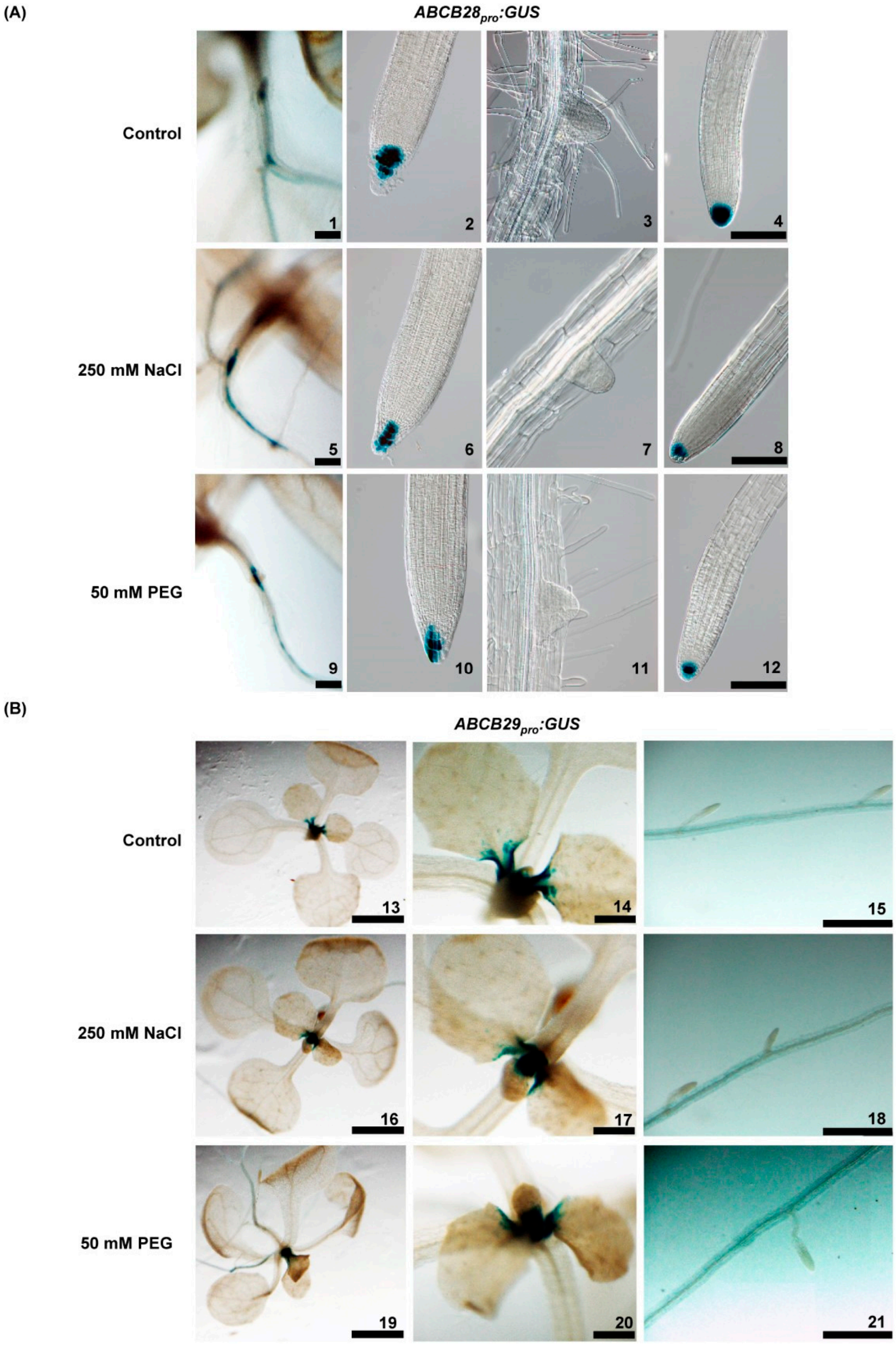

Figure S6. *ABCB28* and *ABCB29* are expressed in cells that produce auxin. Histochemical GUS staining of 2-week-old *ABCB28$_{pro}$:GUS* and *ABCB29$_{pro}$:GUS* seedlings under control conditions or exposed for 5 hours to 250 mM NaCl or 50 mM polyethylene glycol (PEG).

(A) *ABCB28$_{pro}$:GUS* expression in the hypocotyl–root junction (1), root tip (2), and mature but not primordia lateral root tips (3,4). NaCl or PEG stress increased *ABCB28$_{pro}$:GUS* expression in the hypocotyl–root junction (5, 9) but decreased it in root and lateral root tips (6, 8, 10, 12).

(B) On *ABCB29$_{pro}$:GUS* seedlings, GUS staining is prominent on petioles and bases of young leaf primordia (13, 14), as well as in vasculature of the root differentiation zone (15). *ABCB29$_{pro}$:GUS* expression under stress was not affected in these tissues (16–21).

(A, B) Scale bars = 0.1 mm in panels 1, 5, and 9; 0.5 mm in panels 2, 3, 4, 6, 7, 8, 10, 11 and 12; 1 mm in panels 13, 16, and 19; and 0.2 mm in panels 14, 15, 17, 18, 20 and 21.

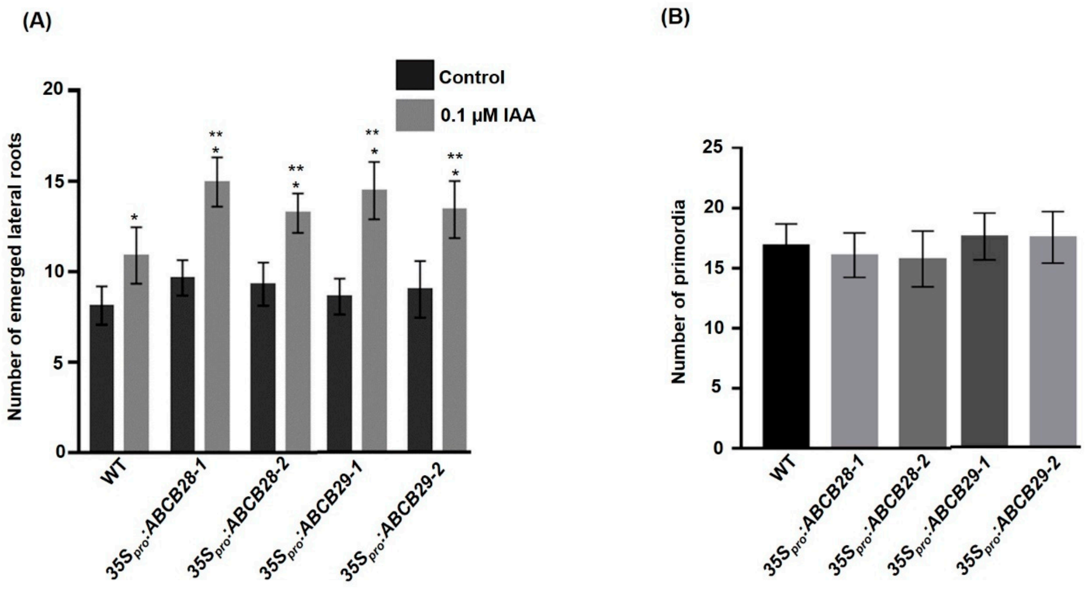

Figure S7. *ABCB28* and *ABCB29* over-expression in plastids alters auxin response. Seven-day-old WT and transgenic plants were treated with IAA. (A) Number of emerged lateral roots were measured after 3 days of IAA exposure- (B) Number of root primordia before IAA addition. Values are expressed as mean ± SD (n = 15 from at least three independent experiments). *Significant differences (Student's t-test; $P < 0.01$) from control conditions. **Indicates significant differences (Student's t-test; $P < 0.01$) from WT.

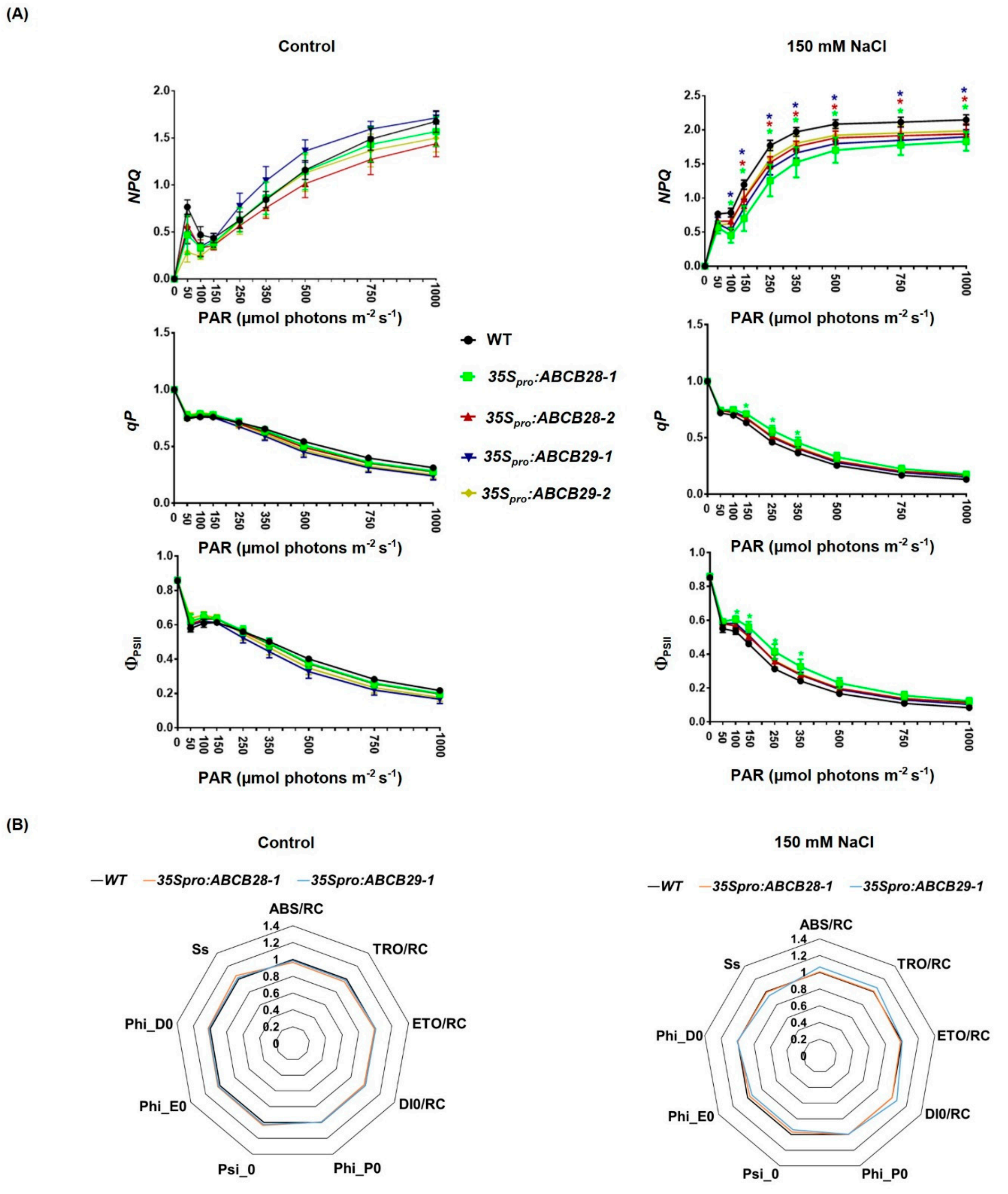

Figure S8. Altered photosynthetic capacity in *ABCB28*- and *ABCB29*- overexpressing plants.

(A) Photosynthetic parameters were determined by measurements of chl*a* fluorescence in whole rosettes from wild-type (WT) and overexpressors grown under long-day conditions for 21 days and then watered with or without NaCl solution for an additional 1 week. Values are means ± SD of 10 plants per genotype from three independent experiments. *Denotes significantly difference from the WT (P ≤ 0.01, Student's *t*-test). *NPQ*, nonphotochemical quenching; *qN*, photochemical quenching; $_{\text{PSII}}$, PSII operating efficiency; PAR, Photoactive radiation.

(B) Fluorescence parameters from OJIP tests using expanded leaves from plants grown as described in (A). Results are presented in radar chart as ratios of values for individual overexpressors and mutants to WT values. Phi_P0, maximum quantum yield of primary photochemistry; Psi_0, probability that a trapped exciton moves an electron into the electron transport chain beyond Q_A^-; Phi_E0, quantum yield of electron transport; Phi_D0, quantum yield of energy dissipation; ABS/RC, absorption flux per reaction center; TR0/RC, trapped energy flux per reaction center; ET0/RC, electron transport flux per reaction center; DI0/RC, dissipated energy flux per reaction center; Ss, single turnover.

Table S1. Effect of salt stress on photosynthetic pigments.

Control

μg/cm²	Chl*a*	Chl*b*	Chl*a* + *b*	Chl*a* : *b*	Carotenoids	Anthocyanins
**WT**	12.72 ± 0.80	5.65 ± 0.47	19.68 ± 1.17	4.52 ± 0.39	1.77 ± 0.20	0.24 ± 0.04
$35S_{pro}$*:ABCB28-1*	12.40 ± 1.36	5.82 ± 0.68	19.55 ± 2.16	4.27 ± 0.24	1.60 ± 0.23	0.24 ± 0.04
$35S_{pro}$*:ABCB28-2*	12.82 ± 0.79	5.67 ± 0.76	19.72 ± 1.33	4.59 ± 0.60	1.75 ± 0.28	0.17 ± 0.05
***abcb28* KD**	10.42 ± 0.87	7.58 ± 1.00	20.22 ± 2.19	2.77 ± 0.24	0.65 ± 0.14	0.27 ± 0.04
$35S_{pro}$*:ABCB29-1*	11.41 ± 1.64	7.53 ± 0.91	20.80 ± 2.77	3.03 ± 0.17	0.77 ± 0.23	0.26 ± 0.04
$35S_{pro}$*:ABCB29-2*	11.24 ± 2.65	6.71 ± 0.84	20.10 ± 3.00	3.39 ± 0.92	1.27 ± 0.90	0.25 ± 0.04
***abcb29* KO**	12.03 ± 1.51	7.98 ± 1.70	22.16 ± 3.66	3.07 ± 0.31	0.80 ± 0.30	0.20 ± 0.01

150 mM NaCl

μg/cm²	Chl*a*	Chl*b*	Chl*a* + *b*	Chl*a* : *b*	Carotenoids	Anthocyanins
**WT**	12.00 ± 1.97	6.72 ± 0.48	20.35 ± 2.56	3.55 ± 0.42	1.21 ± 0.49	0.22 ± 0.05
$35S_{pro}$*:ABCB28-1*	9.93 ± 0.48	7.06 ± 0.57	19.07 ± 1.24	2.82 ± 0.14	0.68 ± 0.07	0.26 ± 0.05
$35S_{pro}$*:ABCB28-2*	11.19 ± 0.95	7.16 ± 0.86	19.98 ± 2.10	3.13 ± 0.13	0.76 ± 0.08	0.18 ± 0.04
***abcb28* KD**	11.98 ± 0.39	5.40 ± 0.41	18.69 ± 1.00	4.45 ± 0.20	1.68 ± 0.05	0.22 ± 0.02
$35S_{pro}$*:ABCB29-1*	11.96 ± 0.83	5.54 ± 0.88	18.78 ± 1.96	4.39 ± 0.41	1.61 ± 0.10	0.23 ± 0.04
$35S_{pro}$*:ABCB29-2*	13.43 ± 2.85	6.77 ± 1.16	21.96 ± 3.35	4.00 ± 0.77	1.70 ± 0.50	0.23 ± 0.17
***abcb29* KO**	13.28 ± 1.14	6.39 ± 0.94	21.82 ± 2.29	4.19 ± 0.30	2.04 ± 0.21	0.23 ± 0.04

Chl*a*, Chl*b*, total chlorophyll content, Chl*a : b*, carotenoids, and anthocyanins were measured from leaf discs (0.5 mm) from 21-day-old wild-type (WT), $35S_{pro}$*:ABCB28*, and $35S_{pro}$*:ABCB29* plants treated with and without 150 mM NaCl for another 1 week. Values are means of 10 replicates (mean ± SD).

Table S2. Extracted and technical parameters from the OJIP protocol. Parameters are expressed per excited cross-section of the tested sample and related to the efficiency of electron transfer downstream from the electro acceptor quinone QA. RC, reaction centre.

**Phi_P0**	**Maximum quantum yield of primary photochemistry**
Psi_0	Probability that a trapped exciton moves an electron into the electron transport chain beyond Q_A^-
Phi_E0	Quantum yield of electron transport
Phi_D0	Quantum yield of energy dissipation
ABS/RC	Absorption flux per RC. Represents the ratio of the total number of photons absorbed at all PSII RCs to the number of active RCs. It is a measure of the PSII antenna size
TR0/RC	Trapped energy flux per RC. The energy is channeled to the RCs where is converted to redox energy by reducing the electron acceptor quinone Q_A while the PSII RC is oxidized, thus creating an electron transport
ET0/RC	Electron transport flux per RC
DI0/RC	Dissipated energy flux per RC. Part of the excitation energy is dissipated in the antenna of the light-harvesting complex II as heat and/or fluorescence.
Ss	The smallest SM turn-over (single turn-over)
F0	fluorescence intensity at 50 μs
FM	maximal fluorescence intensity
Area	Area between fluorescence curve and FM (background subtracted)
SM	area / FM – Fo (multiple turn-over)

Table S3. Primers used in this study.

**Gene name**	**Use**	**Accession no.**	**Primer name**	**sequence (5'-3')**
ABCB28	Entry vector	RAFL21-91-K22	ABCB28-F	ATGGCGTCTGCAACGACTCTTCT
			ABCB28-R	TTACTCAAAGGCTAGTCTCTGAGTGCCAA
			ABCB28-R2	CTCAAAGGCTAGTCTCTGAGTGCCAACA
ABCB29	Entry vector	U10449	ABCB29-F	ATGTCATTTCTCCTCCTAACACC
			ABCB29-R	TCAAATCACGAGTCCAGCTGATGTCAG
			ABCB29-R2	AATCACGAGTCCAGCTGATGTC
ABCB28	GUS constructs		ABCB28-PF	GGTCTCCCCATAAGCAACAAAT
			ABCB28-PR	TGATATTGGTCCAACGAGCTTA
ABCB29	GUS constructs		ABCB29-PF	CTCTCTTTGTTTTGGAATGAATTT
			ABCB29-PR	CACTTTGTACAAGAAAGCTGGGTC
ABCB28	*ABCB28$_{pro}$::ABCB28:GFP* constructs		ABCB28-GF	GGTCTCCCCATAAGCAACAAAT
			ABCB28-GR	CTCAAAGGCTAGTCTCTGAGTGCCAACA
ABCB29	*ABCB29$_{pro}$::ABCB29:GFP* constructs		ABCB29-GF	CTCTCTTTGTTTTGGAATGAATTT
			ABCB29-GR	AATCACGAGTCCAGCTGATGTC
ABCB28	T-DNA insertional mutant	SALK_022541C	LB1-3	ATTTTGCCGATTTCGGAAC
			SALK022-F	GGCAGTAATGTAACTGAGAAACG
			SALK022-R	GGGAGAGTGGATTGAAGAACC-
ABCB29	T-DNA insertional mutant	709C02	08474	ATAATAACGCTGCGGACATCTACATTTT
			709-F	AGATATCTGAATAGCACTGGGCAC
			709-R	GCATTTCATGGTTATGAGTCCAAC
ABCB28	RT-PCR		AtABCB28-F	5′-CCCAATGAGCATGTGTCCAAG
			AtABCB28-R	ACTTGTTGGCACTCAGAGAC
ABCB29	RT-PCR		AtABCB29-F	GCCGTGTCTTCTCATTCCTC
			AtABCB29-R	CTAGTGTCAAGGCAGGACTT
ACTIN 2	RT-PCR		Actin-F	TCGGTGGTTCCATTCTTGCT
			Actin-R	GATCCCATTCATAAAACCCC
